# Comparative analysis, diversification, and functional validation of plant nucleotide-binding site domain genes

**DOI:** 10.1038/s41598-024-62876-5

**Published:** 2024-05-24

**Authors:** Athar Hussain, Aqsa Anwer Khan, Muhammad Qasim Aslam, Aquib Nazar, Nadir Zaman, Ayesha Amin, Muhammad Arslan Mahmood, M. Shahid Mukhtar, Hafiz Ubaid Ur Rahman, Muhammed Farooq, Muhammed Saeed, Imran Amin, Shahid Mansoor

**Affiliations:** 1 National Institute for Biotechnology and Genetic Engineering, College of Pakistan Institute of Engineering and Applied Sciences (PIEAS), Faisalabad, 38000 Pakistan; 2 School of Food and Agricultural Sciences (SFAS), University of Management and Technology (UMT), Lahore, 54000 Pakistan; 3 Department of Life Science, University of Management and Technology (UMT), Lahore, 54000 Pakistan; 4 Department of Biological Sciences, Superior University, Lahore, 54000 Pakistan; 5 Plant Science Division, Research School of Biology, The Australian National University, Canberra, ACT 2601 Australia; 6 Biosystems Research Complex, Department of Genetics & Biochemistry, Clemson University, Clemson, SC 29634 USA; 7 Rheinland-Pfälzische Technische Universität Kaiserslautern-Landau Abteilung Phytopathologie, Paul-Ehrlich-Straße 22, 67653 Kaiserslautern, Germany; 8 Jamil ur Rehman Center for Genome Research, International Center for Chemical and Biological Sciences, University of Karachi, Karachi, 74000 Pakistan

**Keywords:** Genome-wide, *NLR*, Diversity, Land plants, Evolution, Classification, VIGS, Expression, Biotechnology, Computational biology and bioinformatics, Evolution, Molecular biology, Plant sciences

## Abstract

Nucleotide-binding site (*NBS*) domain genes are one of the superfamily of resistance genes involved in plant responses to pathogens. The current study identified 12,820 NBS-domain-containing genes across 34 species covering from mosses to monocots and dicots. These identified genes are classified into 168 classes with several novel domain architecture patterns encompassing significant diversity among plant species. Several classical (*NBS, NBS-LRR, TIR-NBS, TIR-NBS-LRR*, etc.) and species-specific structural patterns (*TIR-NBS-TIR-Cupin_1-Cupin_1, TIR-NBS-Prenyltransf, Sugar_tr-NBS *etc.) were discovered. We observed 603 orthogroups (OGs) with some core (most common orthogroups; OG_0_, OG_1_, OG_2,_ etc.) and unique (highly specific to species; OG_80_, OG_82,_ etc.) OGs with tandem duplications. The expression profiling presented the putative upregulation of OG_2_, OG_6,_ and OG_15_ in different tissues under various biotic and abiotic stresses in susceptible and tolerant plants to cotton leaf curl disease (CLCuD). The genetic variation between susceptible (Coker 312) and tolerant (Mac7) *Gossypium hirsutum* accessions identified several unique variants in *NBS* genes of Mac7 (6583 variants) and Coker312 (5173 variants). The protein–ligand and proteins-protein interaction showed a strong interaction of some putative *NBS* proteins with ADP/ATP and different core proteins of the cotton leaf curl disease virus. The silencing of *GaNBS* (OG_2_) in resistant cotton through virus-induced gene silencing (VIGS) demonstrated its putative role in virus tittering. The presented study will be further helpful in understanding the plant adaptation mechanism.

## Introduction

Gene duplication and loss events are significant drivers of gene family evolution^1^. Duplications result from two primary mechanisms: whole-genome duplication (WGD) and small-scale duplications (SSD). These small-scale duplications include tandem, segmental, and transposon-mediated duplications^2^. These mechanisms seem to represent separate modes of expansion, as gene families evolving through WGDs seldom undergo SSD events, contributing to the maintenance of gene family expansion^3^.

The defense responses mediated by resistance (*R*) genes represent a significant source of resistance against plant pathogens, including viruses^4^. Among the major families of *R* genes, nucleotide-binding leucine-rich repeat (*NLR*) genes are prominent^5^. The Nucleotide Binding Site Leucine-Rich Repeat (*NLR*) gene family originated and diverged into at least three subclasses. Two of these subclasses, each characterized by either a Toll/Interleukin-1 Receptor (TIR) or Coiled-Coil (CC) domain in the N-terminal, play crucial roles as major immune receptors for effector-triggered immunity (ETI) in plants^6^. Simultaneously, the third subclass, distinguished by an N-terminal Resistance to the Powdery Mildew8 (RPW8) domain, functions as a component for signal transfer within this system^7^.

Plant *NLRs,* akin to their animal counterparts, are modular proteins typically comprised of three fundamental components: an N-terminal domain and a central *NB-ARC* domain, which is often referred to as the Nucleotide-Binding Adaptor shared with *APAF-1*, plant resistance proteins, and CED-4. It also contains a C-terminal domain rich in leucine repeats (*LRR*)^8,9^. The central domain of animal *NLRs* is also known as the *NACHT* domain (named after *NAIP, CIITA, HET-E*, and *TP1*)^10^. The *NACHT* domain, while structurally similar to the plant *NB-ARC* domain, exhibits distinct features specific to animal *NLRs*^11,12^.

Plant-*NLRs* exhibit a unique feature with the utilization of either a TOLL/interleukin 1 receptor (*TIR*) domain or a coiled-coil (CC) domain at the N-terminus. This feature distinguishes two major types of plant *NLRs*: the *TIR-*type *NLRs* (*TNLs*) and the CC-type *NLRs* (*CNLs*), respectively^13,14^. Nonetheless, deciphering the structures of N-terminal domains in numerous plant *NLRs* poses a significant challenge. This challenge arises primarily due to their structural variability and the lack of substantial similarity to well-established protein structures^15^. Therefore, *NLRs* with an N-terminus other than the *TIR* domain are occasionally classified as non-*TIR-*type *NLRs* (*nTNLs*). This categorization distinguishes them from *TNLs*.

The *NLR* family has greatly expanded in many plants, resulting in one of the largest and most variable plant protein families^16^. This is in contrast to vertebrate *NLR* repertoires, which typically consist of around 20 members^17^. Many surveyed plant genomes have exhibited large NLR repertoires, and recently, a database, ANNA: an Angiosperm NLR Atlas, was constructed. It contains over 90,000 NLR genes from 304 angiosperm genomes, including 18,707 TNL genes, 70,737 CNL genes, and 1847 RNL genes^18^, similar to the 2012 NBS encoding genes found in wheat^19^.Notably, bryophytes like *Physcomitrella patens* and lycophytes like *Selaginella moellendorffii* represent ancestral land plant lineages^20^. They possess relatively small *NLR* repertoires, with around 25 *NLRs* in the case of *Physcomitrella patens* and 2 *NLRs* in the case of *Selaginella moellendorffii*^21^. This indicates that substantial gene expansion has primarily occurred in flowering plants^22^. Recent research has uncovered that many microRNAs target the nucleotide sequences encoding conserved motifs within *NLRs,* including the P-loop, in a variety of flowering plants^23^. This theory suggests that the comprehensive control of *NLR* transcripts may enable a plant species to maintain extensive *NLR* repertoires without exhausting functional *NLR* loci^24,25^. As microRNA-mediated transcriptional suppression of *NLR* transcripts could potentially offset the fitness costs associated with *NLR* maintenance, this mechanism might contribute to the sustained existence of large *NLR* repertoires^23,26^.

Currently, in Pakistan, almost all cotton varieties of *Gossypium hirsutum* are vulnerable to cotton leaf curl disease (CLCuD)^27^. CLCuD is induced by *Begomoviruses* from the *Geminiviridae* family. The disease is transmitted by the whitefly insect vector, scientifically known as *Bemisia tabaci*^28^. *G. arboreum,* also known as “desi cotton,” represents a high level of resistance against insect pests and diseases including CLCuD, while Mac7 (*G. hirsutum*) is highly tolerant, and Coker-312 (*G. hirsutum*) is highly susceptible to CLCuD^29,30^. *NLR* is the main class of resistance genes that showed a response to viral disease^31,32^. Thus, this study aimed to evaluate the *NBS* domain associated with host plant resistance genes, that might be a potential source of disease resistance genetic elements.

## Methodology

### Genome assemblies and data collection

In the current study, we have selected 39 land plants ranging from green algae to higher plant families including Amborellaceae, Brassicaceae, Poaceae, Citrus, Cucurbitaceae, Malvaceae, Marchaceae, Fabaceae, Nelumbonaceace, Salicaceae, Rosaceae, and Araceae families. In addition, the selection of plants was also made based on ploidy level (haploid, diploid, and tetraploid) for further detailed evolutionary study. The latest genome assemblies (Table S1) were downloaded from publicly available respective genome databases, *NCBI, Phytozome,* and *Plaza* genome databases^33,34^.

### Identification, classification, and comparison among land plants

To screen the *NBS* (*NB-NRC*) domain-containing genes, the *PfamScan.pl* HMM, search script was used with default e-value (1.1e-50) using background *Pfam-A_hmm* model^35^. All genes having *NB-ARC* domain were considered *NBS* genes and filtered for further analysis. In addition, the additional associated decoy domains were also observed through the domain architecture of *NBS* genes by following the Hussain et al.^36^ classification method. In this classification system, similar domain-architecture-bearing genes were placed under the same classes. Furthermore, a comprehensive comparison of classes was also made among land plants.

### Evolutionary study; orthogrouping, and duplication analysis

To provide a deep understanding of the evolution and diversification of *NBS* genes in land plants, we used OrthoFinder v2.5.1 package tools^37^. In this package, the DIAMOND tool was used for fast sequence similarity searches among *NBS* sequences^38^. The clustering of genes was done using the MCL clustering algorithm. The orthologs and orthogrouping were carried out with DendroBLAST^39^. For multiple sequence alignment, MAFTT 7.0 was used^40^. A gene-based phylogenetic tree was also constructed by the maximum likelihood algorithm in FastTreeMP with a 1000 bootstrap value^41^.

### Transcriptomic analyses of *NBS* genes

To ascertain the differential expression and responsiveness of *NBS* genes in various tissues and stresses, we have retrieved RNA-seq data from the IPF database (http://ipf.sustech.edu.cn/pub/)^42^, (Arabidopsis, maize, soybean, upland cotton, and wild cotton) from different databases including Arabidopsis RNA-seq database (http://ipf.sustech.edu.cn/pub/athrna/)^42^, maize RNA-seq database (http://ipf.sustech.edu.cn/pub/zmrna/)^42^, cotton RNA-seq database (http://ipf.sustech.edu.cn/pub/cottonrna/)^42^, soybean RNA-seq database (http://ipf.sustech.edu.cn/pub/soybean/)^42^, Cotton Functional Genomics Database (CottonFGD) (https://cottonfgd.net/)^43^ and Cottongen database (https://www.cottongen.org/)^44^. The Fragments Per Kilobase of transcript per Million mapped reads (FPKM) values were retrieved from respective databases using gene accession as query IDs. The extracted FPKM values the categorized into biotic and abiotic stresses. Besides the FPKM data retrieved from the above-mentioned databases, we also collected additional RNA-seq data from NCBI BioProjects (Bio-projects PRJNA490626 and PRJNA594268, (PRJNA390823) and PRJNA398803)^45–47^. The RNA-seq data is then categorized into three data types; (1) tissue-specific (leaf, stem, flower, pollen, endosperm, pollen, and seed, etc.), (2) abiotic stress-specific (dehydration, cold, drought, heat, dark, osmotic, salt, wounding, etc.) and (3) biotic-stress specific (*Blumeria graminis, Botrytis cereal, Collettrichum tofieldiae, Heterodera schachti nematodes, bacterial strain, Fusarium graminearum,* Rhizotonia *solani, *etc*.*) expression profiling. The RNA-seq data was processed through transcriptomic pipelines, as mentioned by Zahra. et al.^47^. The final heat map was drawn using the TBTool package under heatmap construction with a Log_2_Base value of FPKM^48^.

### Genetic marker prediction in susceptible and tolerant cotton accessions

To find important genetic markers in *NBS* genes, we have selected two contrast accessions of *G. hirsutum* cotton. The Mac7, a highly tolerant *G. hirsutum* to CLCuD, and Coker 312, a highly susceptible accession to CLCuD. The whole genome resequencing data of these plants were collected from NCBI BioProject: PRJNA756435^49^ and PRJNA542238 respectively. The NGS Raw reads were mapped to *NBS* genic regions of the *G. hirsutum* TM-1 reference genome and identified variants (SNPs/InDels). Furthermore, the identified variants were annotated and characterized based on variant type. The variants associated with genes were further compared between the two-accession using the Venny-Bioinfo Tool.

### Gene ontology, KEGG pathways, and *cis*-regulatory elements analysis

To find the functional analysis of *NBS* genes, we performed gene ontology and KEGG pathway enrichment analysis using the Gene Ontology resource and KEGG Pathway Database^50^. The protein features including the number of amino acids, molecular weight, theoretical *pI* (isoelectric point), and Grand Average Hydropathy (*GRAVY*), of *G. hirsutum* were studied using the ProtParam tool–Expasy tool. For the identification of the cis-regulatory elements of *NBS* genes in *G. hirsutum*, we have retrieved 2000b upstream of *NBS* genes and subjected them to *Plant CARE databases*^51^.

### Protein modeling, molecular docking, and target gene mining

To determine the *NBS* protein’s structure and its binding activity to CLCuD viral proteins^52^ and with ATP and ADP molecules, we have used the protein modeling and molecular docking approaches. Based on the biotic stress RNA-seq, we have selected *NBS* genes (upregulated in tolerant; Mac7, while downregulated in susceptible, Coker 312) from wild cotton (*G. arboreum*; naturally resistant to CLCuD) and upland cotton (*G. hirsutum*, susceptible to CLCuD) data. The 3D structure of viral and host proteins was predicted using an *I-TASSER* server with default values^53^. The molecular docking was done using Auto-dock Vina^54^ and MDock web-based server^55^. The 2D and 3D structures were visualized with LigPlus and Discovery Studio, respectively^56^. The binding Gibs free energy was calculated with the ClusPro tool^57^. For further functional analysis, we selected *Gar06G24920* (OG_2_ member), previously reported as a differentially expressed gene under CLCuD in *G. arboreum.*

### Plant seed material

A naturally immune *Gossypium *sp., *G. arboreum* variety FDH-228 was used for the functional analysis of the *NBS* gene. The seeds of *G. arboreum* variety FDH-228 utilized in this study were obtained through a legal and ethical process. The seeds were collected from Gene Isolation Lab, NIBE, Faisalabad, Pakistan by all applicable regulations and guidelines governing the collection and use of plant genetic resources.

### Virus-induced gene silencing (VIGS) protocol

The selected gene (*Gar06G24920_OG*_*2*_) structural features were retrieved from the Cottongen database, and the gene-specific primer was designed with a 500 bp amplicon size of the product. The tobacco rattle virus (TRV) system (pTRV-RNA1 and pTRV-RNA2) was used for VIGS analysis as previously reported by^30^. A 500 bp fragment was PCR amplified from *G. arboreum* cDNA and cloned in a pTRV-RNA2 vector at *Eco*R1 and *Kpn*1 restriction sites. The pTRV-RNA2 clones were confirmed through sequencing. The TRV-GrCLA1 clone was used as a positive VIGS control. The VIGS clones were named TRV: G2, and transformed in *Agrobacterium* strain GV3101 for inoculation. For the VIGS assay, 10 plants of FDH-228 were inoculated for each of the VIGS construct and control at the cotyledonary leaf stage as described by Gao et al.^58^. The inoculated plants were placed at 26 ± 1 °C temperature with a 16/8 h light–dark period in a glasshouse containment facility. After the development of a completely bleached phenotype on TRV-GrCLA1 inoculated cotton plants, gene silencing was analyzed using RT-qPCR and results were compared with the TRV:00 inoculated control plants. The VIGS plants of FDH-228 were inoculated with CLCuD through grafting or by exposure to viruliferous whiteflies. For each condition, five VIGS and control plans were challenged and considered for qPCR-based estimation of virus titer.

#### CLCuD inoculation through grafting and viruliferous whitefly exposure method

To define the function of OG_2_ genes (*Gar06G24920_OG*_*2*_), we have selected a naturally immune *Gossypium *sp, *G. arboreum* variety FDH-228, and used the VIGS approach to silence the selected gene. The silenced plant is then treated with CLCuD through grafting. For graft inoculation, 4–5-inch long CLCuD-infested scions of *G. hirsutum* were grafted with FDH-228 plants. The bottle-neck grafting technique was implemented as described by Akhtar et al.^59^ for successful virus transmission. The diseased scions of *G. hirsutum* were kindly provided and maintained by KP Akhter (PS) at the grafting facility of NIAB. Faisalabad, Pakistan.

The whiteflies were initially collected from the cotton fields of the NIAB. The whitefly culture was maintained on potted cotton plants at 7–10 leaf stages (*G. hirsutum*) in a separate room specified for insect rearing within insect-free cages under the above-mentioned controlled conditions. The maintained culture of whiteflies was assumed to be *B. tabaci* Asia II 1, the most prevalent whitefly species in a cotton-growing zone of Punjab, Pakistan^28,60^. In each cage, both VIGS and control plants were placed side by side and 20–30 adult whiteflies were released per cage. The CLCuD harboring cotton cuttings were placed inside the cages and plants were frequently shuffled for uniform whitefly-mediated virus transmission. The whitefly growth room and glasshouse with grafting experiments were set at 26–28 °C temperature, 68–70% relative humidity, and a 16/8 h light–dark period.

#### RNA extraction and qPCR assay

To assess the VIGS and the viral titer, a total RNA was extracted from VIGS-silenced and control plants. For RNA isolation, leaf tissue samples were taken from the upper three leaves of each plant and RNA was isolated using Trizol (Invitrogen). Purified RNA was used to synthesize cDNA using Revert Aid's first-strand cDNA synthesis kit (Thermo Scientific, USA). RT-qPCR was performed on at least three independent samples of VIGS and TRV:00 inoculated control plants to measure gene silencing. The results were analyzed by the ΔCt method and the 18S gene was used to normalize the corresponding Ct values^30^. Gene-specific primers were used for the expression analysis (Table S33). Twenty-five days post CLCuD inoculation, the leaf discs (~ 3–4 discs/leaf) from the top three leaves of each whitefly exposed and grafted cotton plants were excised and pooled for CTAB-mediated DNA extraction. The purified DNA was nanodroped and diluted to a final concentration of 10 ng/µl. For qPCR analysis and standard preparation previously optimized protocol by Shafiq et al.^61,62^ was followed. The dilutions of plasmids were made in the range from 2, 0.2, 0.02 to 0.002 ng for standard curve preparations.

#### Statement on ethical collection of plant materials

The seed materials utilized in this study are commercially approved varieties and are readily available in the market. Specifically, *Gossypium arboreum* variety FDH-228 was sourced from the Gene Isolation Lab, NIBGE, Faisalabad, Pakistan. All requisite permissions and licenses for specimen collection have been acquired, in strict adherence to regulatory guidelines. This study is committed to maintaining ethical standards, respecting intellectual property rights, and ensuring transparent documentation of the sources throughout the research process.

## Results

### Genome-wide identification

**Figure 1:**
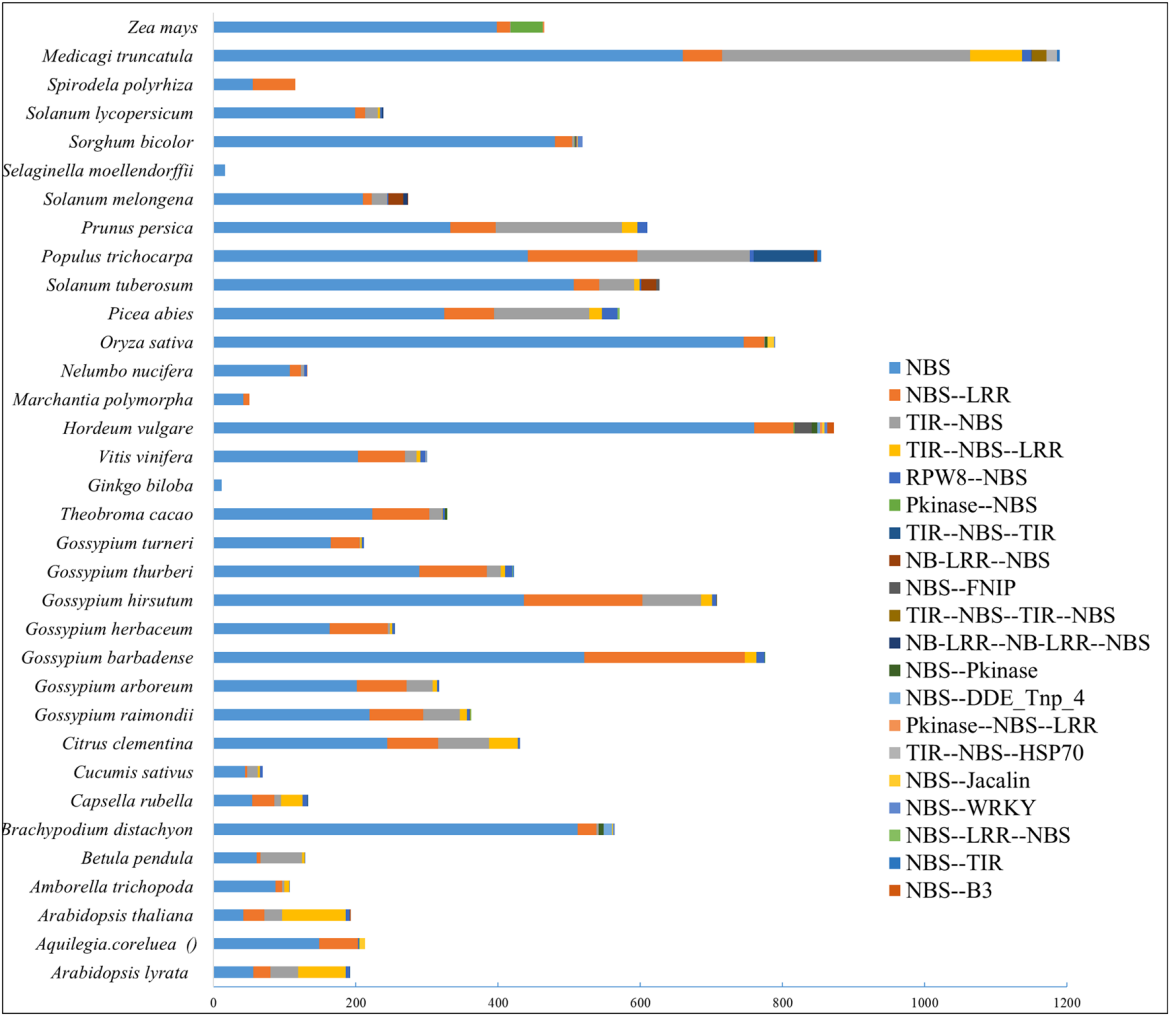
Genome-wide identification and classification of NBS genes in Land plants. The length of the bar represents the total number of genes and different colors demonstrate the top 20 subclasses, based on the domain architecture.

The whole-genome screening analysis identified a total of 12,822 *NBS*-encoding genes across 34 species. The species-based genome-wide identification demonstrated that the number of genes present in each genome was independent of its genome size. The genome size to the number of *NBS* gene regression (R^2^) values was observed as below the cutoff value (R^2^ = 0.015). Interestingly, we observed a correlation of genome size to the number of *NBS* genes in *Gossypium* sp. (R^2^ = 0.669). For instance, *G. hirsutum* with a genome size of 2.4 Gb has the highest number of *NBS* genes (708 *NBS* genes), followed by *G. barbadense* (622 *NBS* genes) with a genome size of 2.3 Gb, and *G. arboreum*, a diploid genome with a 1.7 Gb genome, has 365 *NBS* genes. Similarly, *G. raimondii* with a 0.7 Gb genome has 323 *NBS* genes (Fig. 1, Tables S1 and S2).

### Classification of *NBS* genes in land plants

The *Pfam* domain analysis identified several decoy domains in addition to the *NBS* domain. The deep analysis of domain architecture reported several functional protein domains associated with *NBS* domains (Table S2). So, based on the conserved domains, motifs, and their sites in the primary sequences of *NBS* proteins, all 12,822 genes were divided into 168 classes concerning the presence /absence and copy number of associated domains (Table S3). Of these classes, the 33rd class (only the *NBS* domain) has 8591 *NBS* genes, showing the largest class of *NBS* superfamily and most of the classes were highly specific with few numbers of *NBS* genes. So, based on the observation, it can be estimated that the main superfamily of *NBS* proteins contains only the *NBS* domain. The species-wise distribution and diversity of identified *NBS* classes were also interesting. As the lower plants possessed only either one or two specific classes e.g., *Selaginella moellendorffii* belong to a lycophyte, and possessed only 33th classes (only *NBS* single domain) with 16 *NBS* genes. Similarly, *Spirodela polyrhiza* is a species of duckweed known by the common name common duckmeat and has only two classes 33rd and 60th (*NBS* and *NLR*). In contrast, the evolutionary development and adaptation process causes drastic changes in the genome of higher land plants. As moved from simple to complex plants, the *NBS* classes became more complex. For instance, *Hordeum vulgare*, barley, a member of the grass family, is a major cereal grain grown in temperate climates globally and has the highest number of *NBS* classes and several unique *NBS* domain architectures. Similarly, *Vitis vinifera*, the common grape vine, a species of flowering plant, native to the Mediterranean region, also has twenty *NBS* classes. Among all land plants, the most common domain architectures were detected as *NBS, NLR, TIR-NBS, TIR-NBS—LRR*, and *RPW8—NBS* domain architectures. However, we also observed highly species-specific classes like *Methyltransf_11—NBS, NBS-Glyco_transf_8, TIR-NBS-Lectin_legB-Pkinase, TIR-NBS-RHD3,* and *NB-LRR-NB-LRR-NBS-Retrotran_gag_2*. Overall, out of 168 classes, 94 classes were specific-specific, some classes were family-specific, and were genus-specific like the Gossypium species showed a close relationship regarding the classes. The tetraploid species (*G. barbadense* and *G. hirsutum*) of *Gossypium* sp. have more diversity rather than other diploid species (Fig. 1, Tables S3 and S4).

### Orthologs analysis: orthogrouping, duplication, and overlapping,

The orthologs groping analysis of 12,822 *NBS* in 33 land plants demonstrated that 91.8% of total *NBS* proteins were assigned orthogroups. However, only 8.2% of *NBS* proteins were highly unique and could not fit into any orthogroups. A total of 664 orthogroups were predicted and out of these, 347 orthogroups (holding 1135 *NBS* proteins) were species-specific orthogroups and the mean and median orthogroup size was 18 and 3 genes, respectively (Table S5). The species-based comparative genomics overexposed the genetic makeup of different land plants regarding *NBS* genes. We identified many common/core genes as well as species-specific unique genes based on sequence divergence. For instance, in the case of *Arabidopsis layrata*, a total of 209 *NBS* genes were predicted and categorized into 37 orthogroups (with 2 species-specific orthogroups) covering 206 *NBS* genes. The remaining three genes did not fit any orthogroups due to their clear sequence divergence as compared to other *NBS* genes. Similar results were also observed for other land plants. For instance, in the *Gossypium *sp. the *Gar, Gba, Ghi, Ghe, Gth, and Gra* did not show any species-specific unique orthogroup or ortholog. But observed some unique *NBS* genes were categorized as “unassigned orthogroup genes” (Table S6). The number of genes in orthogroups of each species was also assessed and identified that the “OG_000_” orthogroup possessed the highest number with 2535 *NBS* proteins and shared all species except some lower plants including *Gbi, Mpo*, and *Smo*. In addition, some orthogroups were highly specific to species like OG_400_ was only present in *Al* with *ALNBS134* and *ALNBS135* genes. Similarly, the OG_024_ was specific to *Mtr* with more than 15 *NBS* genes and the OG_454_ (*GhirNBS444* and *GhirNBS445*), and OG_455_ (*GhirNBS626* and *GhirNBS627*) were specific to *G. hirsutum.* Similar unique genes were also identified in other land plants (Tables S7, S8, S9 and S10). The orthogroups duplication event analysis conformed several duplication events during the evolutionary process e.g., the OG_000_ duplicated 1932 times with 100% confidence and 1600 times with a 50% confidence level. Similarly, other orthogroups were also passed by some duplication events (Tables S11 and S12).

To find the relationships among different species the orthogroups and orthologs overlapping were also assessed and compiled into clusters using dendrogram. The result demonstrated that *Osa, Hvu, Zmy, Bra,* and *Sbi* formed one clade, and *Sly, Stu, and Sme* formed another clade. Similarly, the *Gossypium* sp. Including *Gth, Gtu, Gra, Gba, Ghi, Gar*, and *Ghe* formed one clade. In addition, the *Gar* (A-genome) and *Ghe* (A2-genome) shared sister branches, and the *Ghi* (AD-genome), and *Gba* (AD2-genome) formed another sister branches showing the highest genome similarity index (Figs. S1, S2, and Table S13).

### Phylogenetic tree analysis: duplication events at nodes and gene-based phylogenetic tree

**Figure 2:**
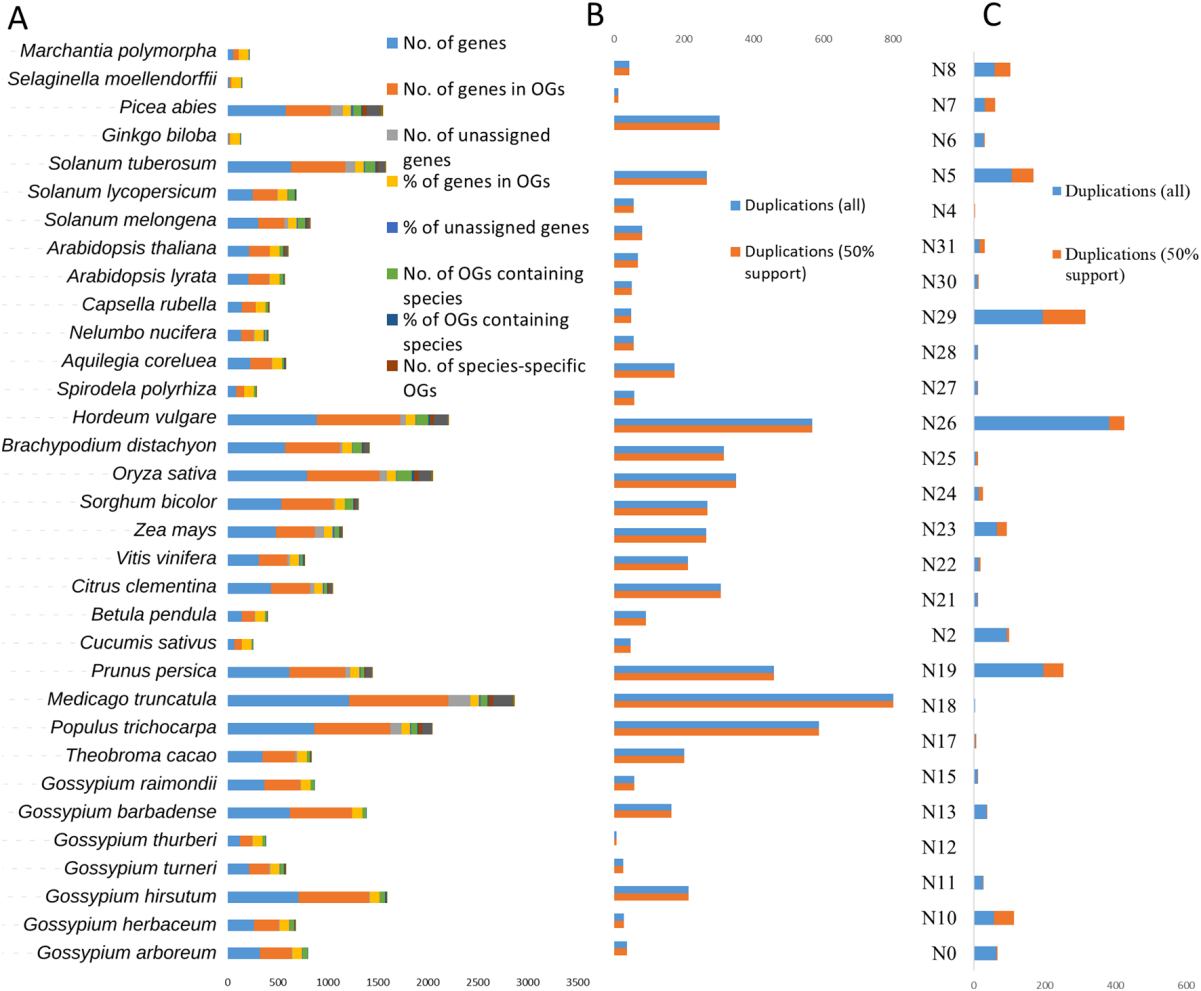
Evolutionary study of NBS genes in land plants. (**A**) Basic statistics of orthogroups with abbreviated species names. (**B**) Gene duplication events at terminal nodes of the phylogenetic tree, and (**C**) gene duplication events at internal nodes of a phylogenetic tree.

The duplication events at internal and terminal nodes indicated the evolutionary relationship of *NBS* genes in land plants. The duplication events at terminal nodes demonstrated that *Ginko biloba* was highly conserved and did not demonstrate any duplication at the terminals as compared to other land plants. Entering the *Gossypium* genus, the N_24_ node further duplicated more than 210 times and separated other *Gossypium* sp. from *G. raimondii* (Table S14). In conclusion, gene duplication events at internal and terminal nodes demonstrated the expansion of the *NBS* gene family from lower plants to higher plants. The duplication events also cause the gain and loss of NBS-associated terminal domains, as we observed in the domain architecture analysis. This diversity in *NBS* proteins might be an adaptation of land plants (Fig. 2).

The gene-based phylogenetic tree was divided into 282 sub-clusters and each cluster was correspondence to the orthogroups. The highest number of genes was observed in cluster OG_000,_ which gradually decreased to and decreased to OG_282._ In addition, some clades were highly species-specific, and some were genus while very few were family specific. Due to the large number of genes in top orthogroups only OG2 was present in the phylogenetic tree (Fig. S3).

### RNA-based expression profiling

**Figure 3:**
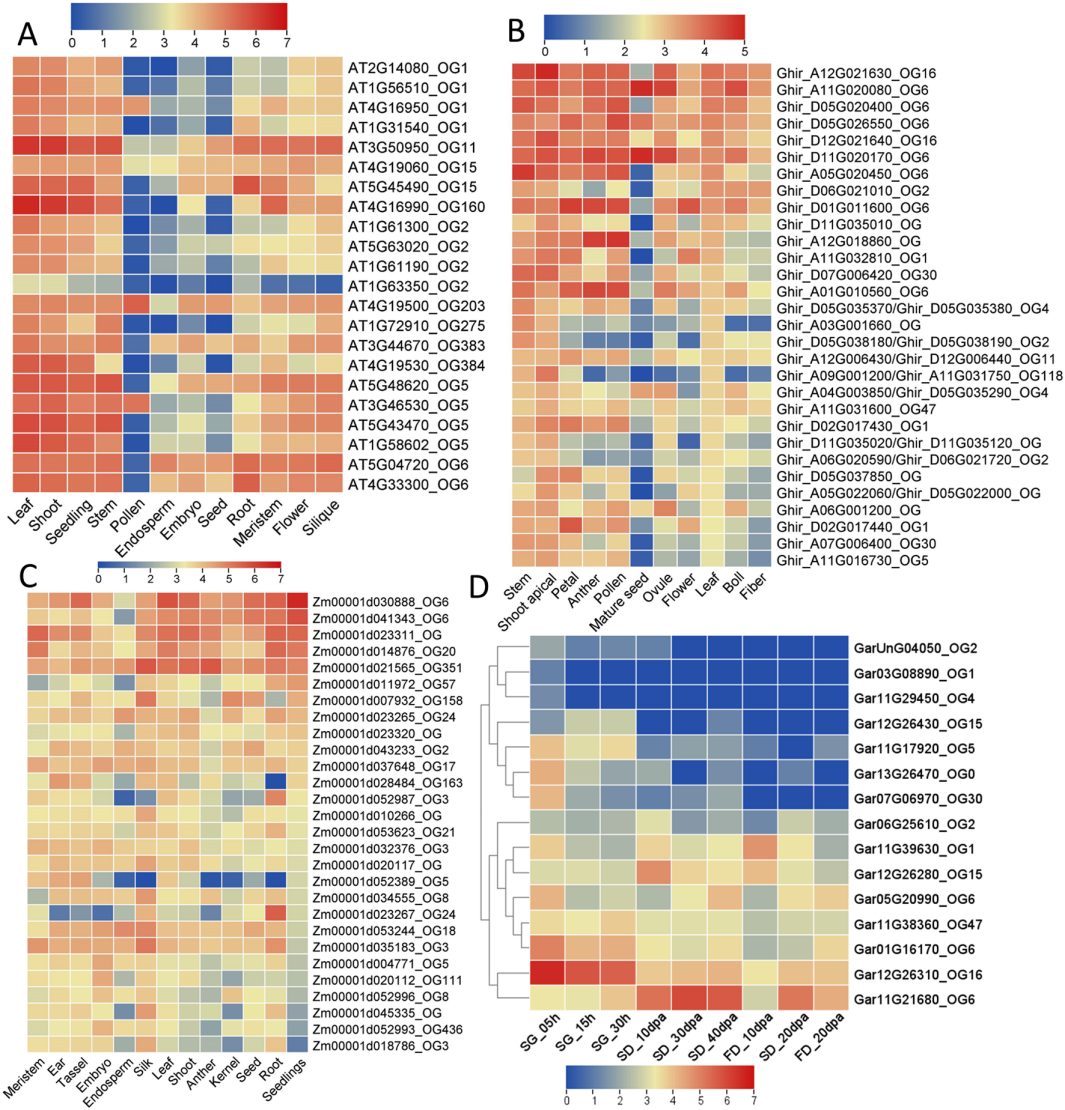
RNA-seq-based expression profiling of NBS gene in different tissues. The OGs associated with gene IDs represent the orthogroups. (**A**) *A. thaliana*, (**B**) *G. hirsutum*, (**C**) *Z. mays* and (**D**) *G. arboreum*.

For a deep understanding of *NBS* genes in different tissues under different biotic and abiotic stresses, we have selected three plant species including *A. thaliana, Z. mays, G. arboreum,* and *G. hirsutum*. Based on the RNA-seq expression data most important putative genes were further filtered for detail study. The tissue-specific expression profiling of *NBS* genes in *A. thaliana* generally demonstrated that most of the genes are differentially expressed in leaf, shoot, seedling, flower, and silique. However, very little or negligible expression is observed in pollen, endosperm, embryo, and seed. At the orthogroups (OGs) level, some OGs were highly specific to some tissues like OG_6_ (*At4G33300*_*OG6*_ and *At5G04720*_OG6_), OG_15_ (*AT5G45490*_OG15_) had the highest expression in root tissue (Table S15). Similarly, the OG_2_ (*AT1G61300*_OG2_, *AT5G63020*_OG2_, and *AT1G61190*_OG2_) showed similar patterns in leaf, shoot, and seedling. The cladogram among tissues and genes also demonstrated a co-expression network of genes. In the case of *Zmy* tissues, three major co-expression pattern networks were observed among *NBS* genes (Fig. 3, Tables S16, S19, S22 and S25).

The RNA-seq data of abiotic stresses included oxidative stress, UV, nutrient deficiency, dark, salt, cold, drought, heat, ozone, and wounding stresses. Generally, it was observed that the OG members mostly co-expressed under various abiotic stresses. In *A. thaliana*, the *NBS* genes are most commonly expressed in all stresses except oxidative and UV stresses. Among OGs, the OG_6_, OG_11,_ and OG_1_ showed similar expression patterns with the highest expression under most of the stresses. Similarly, the OG_6_, OG_0,_ and OG_351_ formed one co-expression clade in the *Z. mays* plant under various abiotic stresses (Fig. S4, Tables S17, S20 and S23).

**Figure 4:**
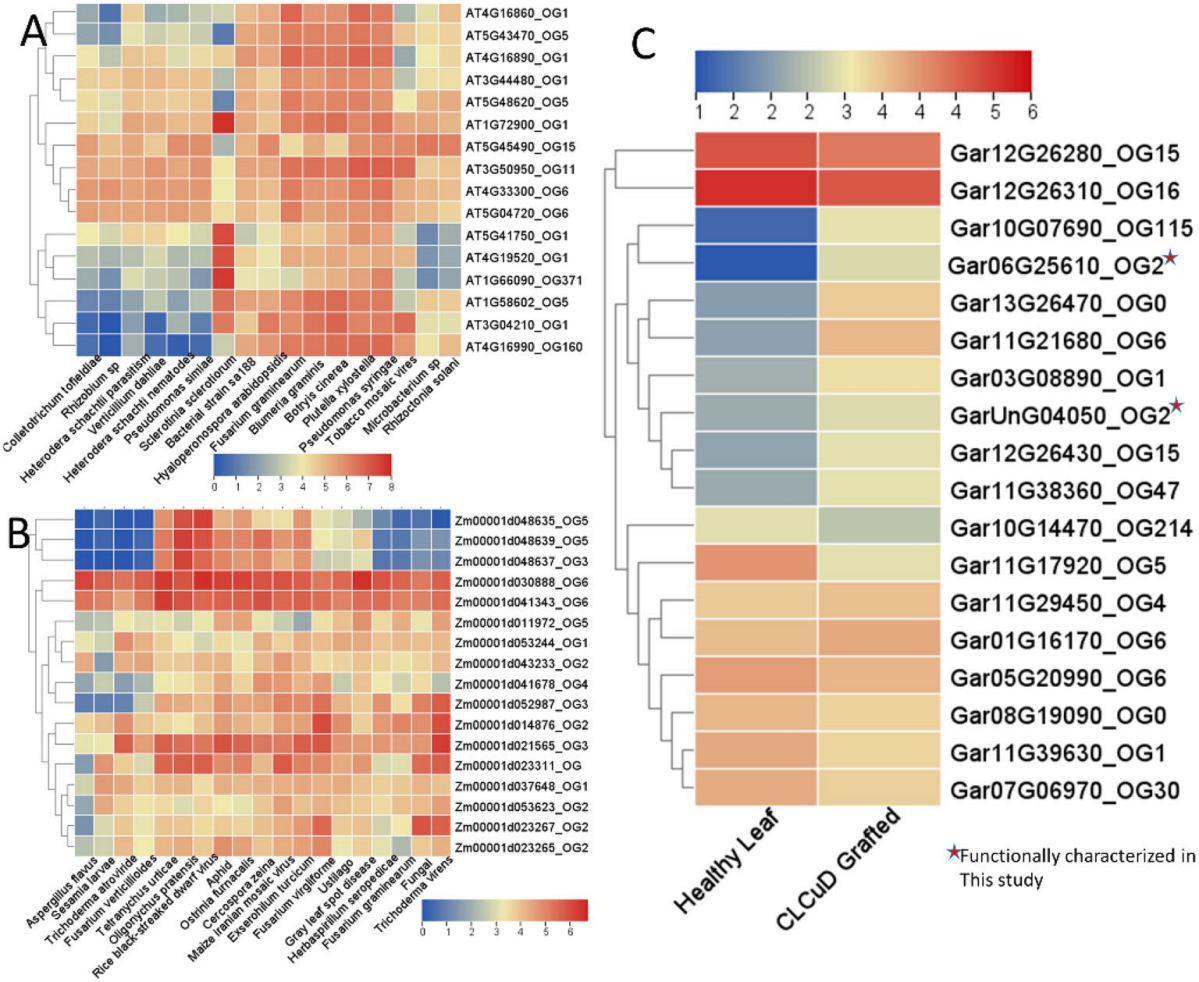
RNA-seq based expression profiling of NBS gene under different biotic stresses. The OGs associated with gene IDs represent the orthogroups. (**A**) *A. thaliana*, (**B**) *Z. mays* and (**C**) *G. arboreum.*

**Figure 5:**
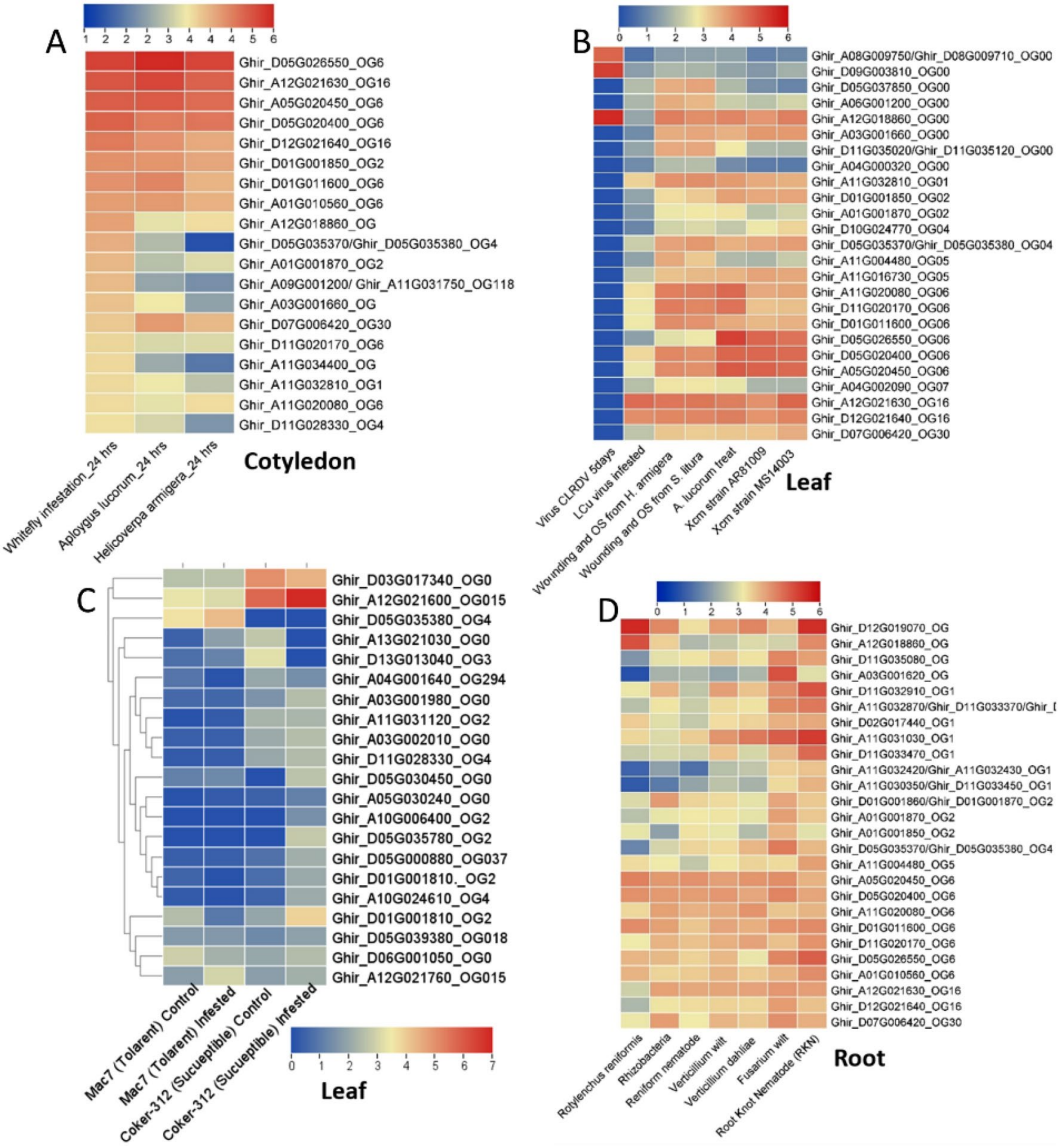
Expression profiling of *G. hirsutum* NBS genes in different tissues under various biotic stresses. (**A**) Cotyledon tissues, (**B**) leaf tissues, (**C**) expression of NBS gene in two contrasting accessions, 1; Mac7 (tolerant to CLCuD), 2; Coker312 (highly susceptible to CLCuD) under CLCuD infection in leaf. (**D**) under various root-associated pathogens in root tissue.

The biotic stress expression profiling included different pathogens from aphids to viruses (aphids, nematodes, fungi, bacteria, and viruses). The analyses identified several *NBS* gene responses under different pathogenic stresses in *Arabidopsis, Z. mays, G. arboreum,* and *G. hirsutum*. For instance, the OG_1_ (*AT1G66090*_OG1_*, AT4G19520*_OG1_*, AT5G41750*_OG1_*, and AT1G72900*_OG1_) significantly upregulated under *Slerotinia sclerotiorum* (Fig. 4A). Similarly, under viral disease, the OG_1_, OG_5_, and OG_6_ showed putative responses during viral infection in Arabidopsis and *Z. mays* (Fig. 4B). The *G. arboreum* is naturally resistant to several viral diseases, so we have taken the CLCuD grafted RNA-seq for the identification of the *NBS* gene’s role in the presence of viruses. We identified several differentially expressed *NBS* genes in *G. arboreum* under grafted-CLCuD. The OG_2_ (Gar06G25610_OG2_, GarUnG04050_OG2_), OG_6_ (Gar11G21680_OG6_), OG_4_ (Gar11G229450_OG4_) and OG_115_ (Gar10G07690_OG115_) showed significantly upregulation under CLCuD (Fig. 4C). The Gar06G25610_OG2_ was further validated through gene silencing approaches in this study (will discuss later) (Fig. S5). As we are so interested in the identification of most putative *NBS* genes and their role in *G. hirsutum* under cotton leaf curl disease, which is one of the major challenges in Pakistan, we presented a two-contrast agronomic trait associated accession (Mac7 (a highly CLCuD tolerant *G. hirsutum* accession developed by USDA, but low production) and Coker312 (highly susceptible, most regenerative accession, commonly used for tissues culturing, also posseted important agronomic traits) RNA-seq data. The results were surprising, we found differential regulation of *NBS* genes in tolerant accession (Fig. 5, Tables S18, S21, S24 and S26). In summary, we found that the OG_0_, OG_2_, OG_5_, OG_6,_ and OG_15_ have a highly responsive role in different tissues and under various biotic and abiotic stresses in *Arabidopsis, Z. mays*, *G. arboreum* and *G. hirsutum*.

### Variants detection in tolerant and susceptible accessions

**Figure 6:**
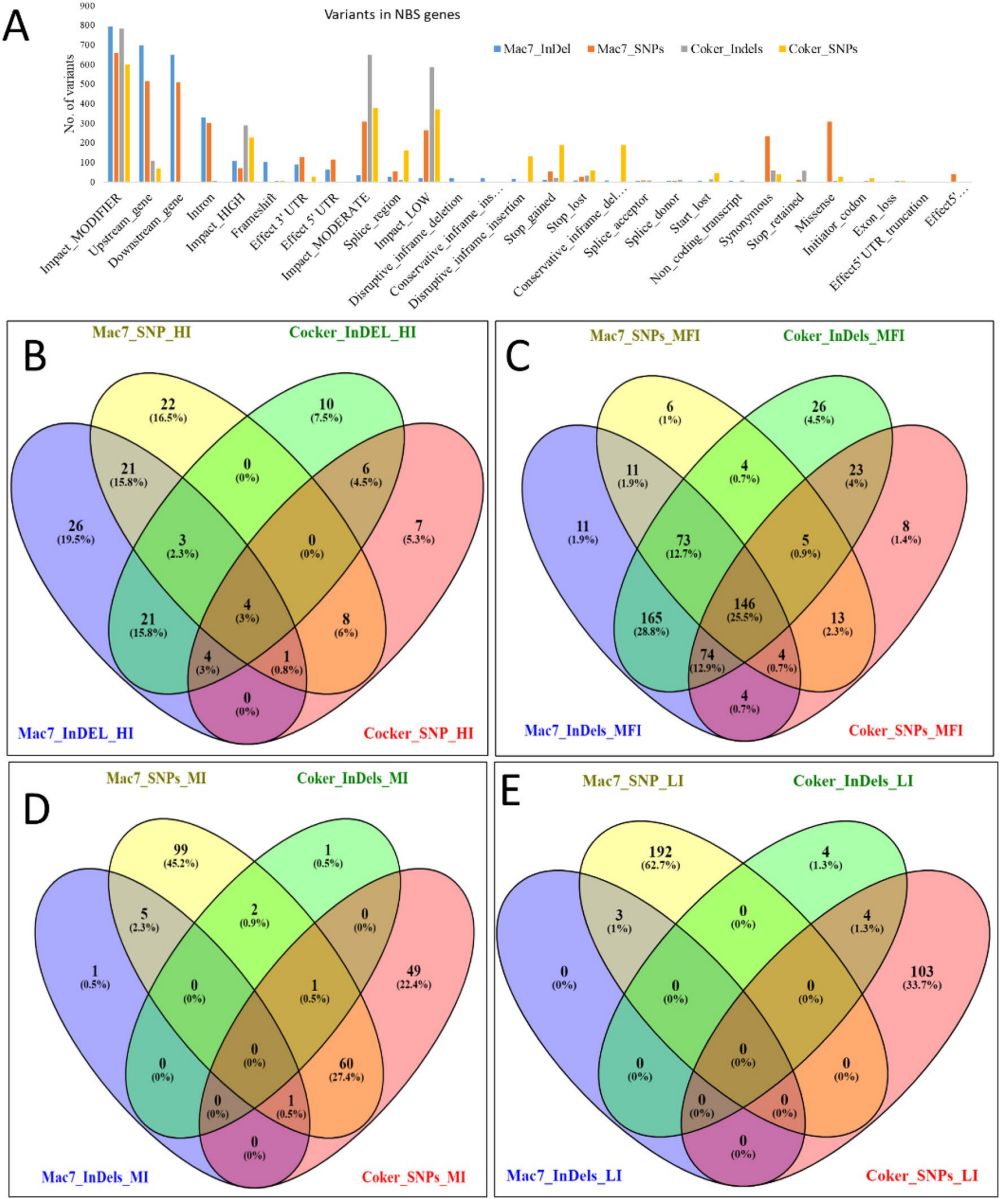
Genetic variations of NBS genes between Mac7 and Coker 312. (**A**) variants distribution region Based on impact level of variants on gene functions. (**A**) HI; High Impact, (**B**) MFI; Modifier Impact, (**C**) MI; Moderate Impact, (**D**) LI; Low Impact, comparison at SNPs (single nucleotide polymorphisms) and InDels (Insertion and Deletion).

The genome-wide genetic diversity of Mac7 (*G. hirsutum* tolerant) and Coker312 (*G. hirsutum susceptible*) with reference to TM-1 *G. hirsutum* reference genome, identified several SNPs and InDels in *NBS* genomics regions of Mac7 (InDels: 2989, SNPs: 3594) and Coker 312 (InDels: 2646, SNPs: 2527). The identified variants were characterized into four impact levels *i.e.,* impact as high (affecting splice-sites, stop and start codons), moderate (non-synonymous), low (synonymous coding/start/stop, start gained), and modifier (upstream, downstream, intergenic, UTR). A comparative study of high-impact SNPs and InDels associated genes between Mac7 and Coker identified several unique and common variants e.g., Coker 312 (7 SNPs, 10 InDels) and Mac 7 (22 SNPs, 26 InDels) unique variants. While 4 genes were common between the two accessions. Similarly, the modifier and moderate and low-impact variants were also observed in the two accessions (Fig. 6, Tables S27, S28 and S29).

### Physiochemistry, gene ontology, and metabolic pathways

The amino acid (AA) sequence analysis of *Gossypium hirsutum* showed a length ranging from 500 to 1500 amino acid (AA), molecular weight (MW) ranged from 50 to 150 kDa, isoelectric point (pI) value ranged from 2 to 6, charge ranged from + 55.5 to − 44.5 and the Grand Average of Hydropathy ranged from 0 to − 0.2. The gene ontology comprises three components; biological process, molecular function, and cellular components. In the case of molecular function, the results demonstrated that the *NBS* is mainly involved in the Adenosine diphosphate (ADP) and Adenosine triphosphate (ATP) binding activity with signal transduction. The KEGG pathway analysis indicated the role of the *NBS* gene in plant-pathogen interaction (PPI) and signaling pathways (SP). The promoter analysis identified several stress-responsive elements in the promoter region of NSB genes. A total of 72 cis-regulatory elements were identified some of the responsive elements were present in all *NBS* genes like *TATA-box, CAAT-box, Box, ARE, G-box, GT1-motif, ABRE, CGTCA-motif, TGACG-motif, TCT-motif, TCA- element, MBS, GATA-motif, CAT-box*, and *O2*-site. Most of the cis-regulatory elements belonged to stress responses (Fig. S5, Table S30).

### Molecular docking, and protein–protein interaction

For the proteins modeling, we selected genes based on upregulated in *G. arboreum* (naturally resistant to CLCuD) under grafted-CLCuD and whitefly induced in Mac7 (*G. hirsutum*, tolerant to CLCuD) covering OG_0_, OG_2_, OG_15_, and OG_43_. The selected genes’ translated proteins were used for 3D structure prediction using the *I-TASSER* server. The PDB database *proteins 6J5T-C,6S2P-N, 7CRB-A,4TZH-A,* and *4U09-A* were used as template sequences. The 3D molecules of ATP and ADP were downloaded from the cheminformatics database. The docking results demonstrated a stable interaction of *NBS* genes with ATP and ADP with a range of − 7 kcal/mol to 8.2 kcal/mol, except *Gar09G25760_OG*_*43*_ and *Ghir_A12G021600_OG*_*15*_, which showed below − 6.8 kcal/mol and − 6.6 kcal/mol, respectively. The binding affinity of *Gar12G23120_OG*_*0*_ showed that it has a more stable interaction with ATP as compared to the ADP molecule. Similarly, the OG_2_ genes (*Gar01G01860*_OG2_*, Gar06G24920*_OG2_) showed more affinity to the ATP molecule as compared to the ADP molecule. In contrast to this, the OG0 (*Gar11G29700*_OG0_*, Ghir_D13G021900*_OG0_) showed more stable interaction with the ADP molecule. The interacting residues of *NBS* proteins varied from ATP to ADP e.g. the ATP binds with Lys179, His240, Trp139, Val261, Gln 264, Glu142, and Val 284, whereas the ADP binds with Asp143, Glu142, Asn289 and Lys286 in Gar12G23120_OG_0_ protein. Similarly, other binding results also demonstrated similar patterns (Fig. S6, Table S31). For further detailed molecular interaction of OG_2_ genes (*Gar06G24920_OG2*) with CLCuD viral proteins (AC1, AC2, AC3, AC4, AV1, and AV2), we assessed the interaction level by Gibs free energy. The *Gar06G24920_OG*_*2*_ protein demonstrated the highest stability value (− 1352 kcal/mol) with AC1 viral proteins followed by AV2 (− 1103.8), AV1 (− 1026.4), and so on. Similarly, another member of OG_2_ group protein (*Gar06G25610/Gohir.A06G19220/Ghir_A06G020580*), also demonstrated high interaction value with AC1 (− 1506.6 kcal/mol) and AV1(− 1255.8 kcal/mol), AC3 (− 1230.8 kcal/mol). The active residues were also changed with the viral protein complexes. (Fig. S7, Table S32).

### Silencing of OG_2_ (G2) genes enhanced virus titer in *G. arboreum*

**Figure 7:**
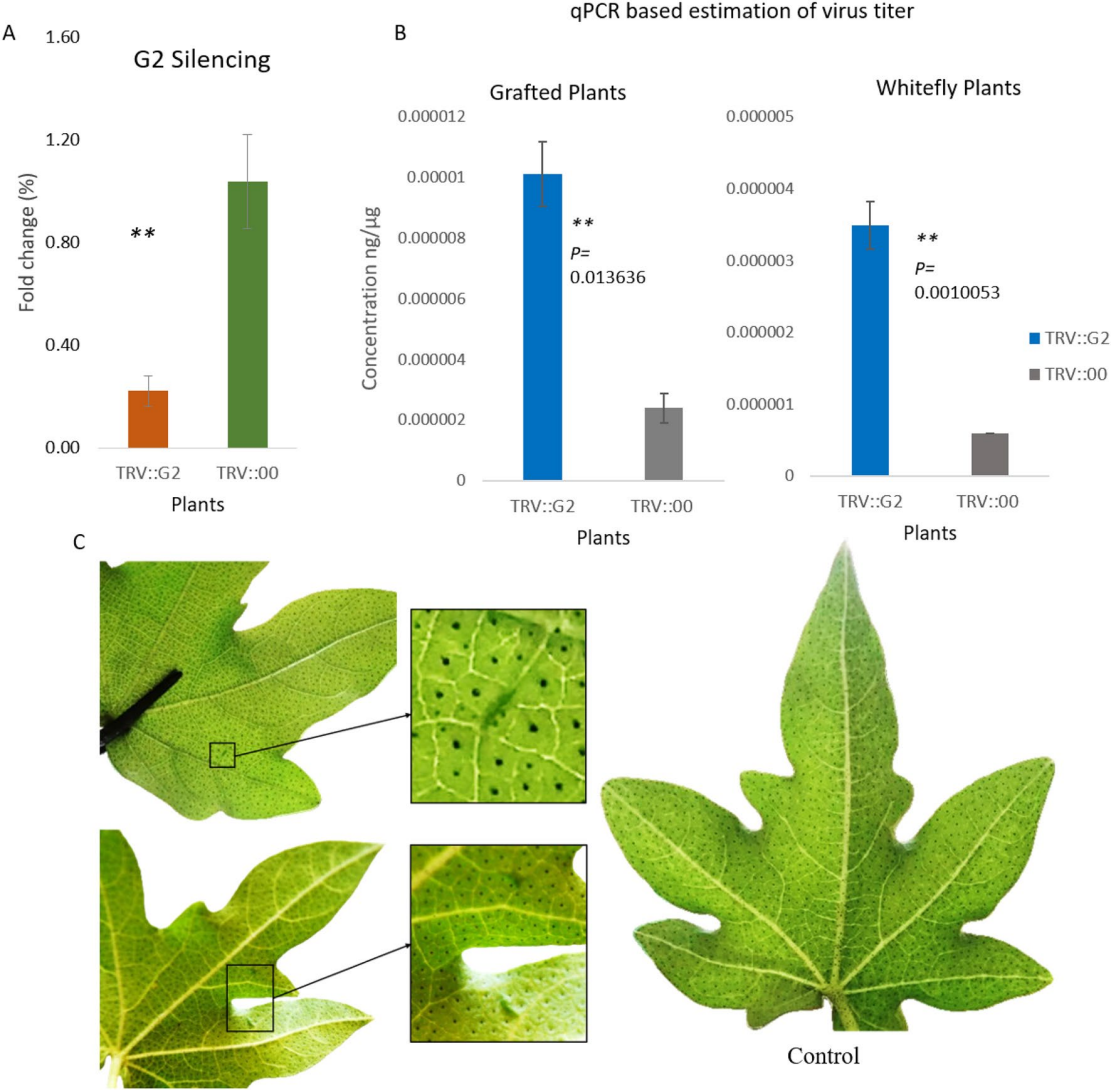
qPCR-based estimation of gene expression and CLCuD Symptoms development on VIGS plants. Panel** (A**), Orange represents a decreased expression of the G2 gene in *G. arboreum* plants. Green shows showing expression of G2 in empty vector inoculated control plants. Typical CLCuD symptoms of lower grade appeared on G2 VIGS plants. Panel (**B**) shows qPCR-based estimation of virus titer in both graft and whitefly exposed VIGS plants of *G. arboreum.* ** is for a significant difference. Panels (**C**) is showing minor vein thickening on G2 VIGS plants and TRV:00 inoculated FDH-228 plant leaf with no symptoms. The plants were picturized using a microlens-assisted camera of the Apple iPhone 11 pro max model a2161.

TRV-based VIGS assay in cotton is a well-established approach to conducting functional studies of OG_2_ (*Gar06G24920_OG2)genes*. FDH-228 plants were inoculated with VIGS constructs to silence the subjected genes, 15 days post infiltration, a completely bleached phenotype was visible on TRV-GrCLA1 inoculated cotton plants, at this time, RT-qPCR based gene silencing was performed (Figs. S8, S9). The results demonstrated a considerable reduction in the expression level of the G2 gene in FDH-228 silenced plants, compared to the TRV:00 inoculated control plants (Fig. 7A). The graft-mediated CLCuD inoculation has been well demonstrated by Ullah et al.^58^ in *G. arboreum* plants. Since this breakthrough, it has become possible to identify the resistance imparting genes of *G. arboreum* against CLCuD*.* After assessing the gene silencing, an equal number of silenced and control plants were inoculated with CLCuD by grafting CLCuD harboring scions, and exposing them to viruliferous whitefly. 25 days post-CLCuD exposure, enhanced virus concentration was witnessed in G2 (*Gar06G24920_OG2*) silenced plants in comparison to TRV:00 inoculated controls both under graft and viruliferous whitefly exposure (Fig. 7B). Minor disease symptoms were evident only on graft-inoculated G2 (*Gar06G24920_OG2*) silenced plants (Fig. 7C). These results suggested the likely involvement of the G2 (*Gar06G24920_OG2*) gene in CLCuD resistance in FDH-228 plants.

## Discussion

In the last two decades, several types of resistance (*R*) genes have been discovered. The majority of R genes found so far are members of the nucleotide-binding site (*NBS*)-leucine-rich repeat (*LRR*) receptor (*NBS-LRR*, also known as *NLR*) gene family^63^. *NLR* genes have been found in plants and their origin may be traced back to the common ancestor of all green plants. Before the split of green plants, phylogenetic studies revealed that *NLR* genes had diverged into separate subclasses^20,64^. According to genome-wide studies, different species of plants possess a varying number of *NLR* genes. It was also observed that the number of gene was correspondence to the ploidy level of plant Species like the tetraploid cotton, *Gossypium hirsutum* (AD1), and *Gossypium barbadense* (AD2), had a higher number of NBS genes compared to their progenitors *G. arboreum* (A2), *G. herbaceum* (A1), and *G. raimondii* (D5) (diploid species). Similar results were also observed in other plant species; for instance, bread wheat (*Triticum aestivum*), a hexaploid, possessed 2012 NLRs^19^., while its progenitor wild species, *Aegilops*, had half of the detected wheat, with 742, 800, and 1030 candidate NLRs in *Ae. longissima*, *Ae. sharonensis*, and *Ae. speltoides*, respectively^65^.Additionally, the simpler genome had fewer NBS candidate genes, like Jia-Yu et al. identified 43 *NLR* in *Marchantia polymorpha*^66^ while our study listed 54 *NLR* (this study) in the same plants and similar contrast was also found in a few other plants like 443 (literature)^67^, and 636 (this study) in *Solanum tubersum.* A list of detailed comparisons of *NBS* from the literature and this study give significant differences in the number of *NBS* genes (Table S34). The variation in this study and previous ones might be due to the use of the latest genome assemblies as well as the improved algorithms, which was the main focus of this study. Furthermore, we have also observed that some of the green plant ancestors like *Coccomyxa subellipsodiea, Klebsormidium flaccidium, Micromonas pusilla, Physcomitrella Patens,* and *Volvox carteri* do not possess *NBS* genes, indicating the lack of such resistance gene in ancestor plants. *NLR* gene expansion and contraction can support the “birth-and-death hypothesis” that predicts fast *NLR* evolution in land plants, which might be due to duplication events at the genome, and segmental levels^68,69^.

*NLR* genes are frequently clustered in complex clusters, a structure that may favor *NLR* dynamic development and diversification to cope with rapidly evolving pathogens^70^. The detection of certain pathogen effectors activates *NLRs,* resulting in a robust immune response that is frequently linked with localized programmed cell death, known as the hypersensitive response (HR)^71^. Most *NLRs* feature an N-terminal extension consisting of a Toll/interleukin-1 receptor (*TIR*) domain, a coiled-coil domain (CC), or a divergent coiled-coil domain (CCR) identical to the Resistance to powdery mildew 8 (*RPW8*) domain^72^. Generally, there are three kinds of *NLRs* based on the N-terminal domain and evolutionary history of the *NB-ARC* which are (1) *TIR-NLRs* (*TNLs*), (2) CC-*NLRs* (*CNLs*), and (3) *RPW8-NLRs* (*RNLs*)^15,73^. Our classification system was unique and identified several N-terminal and C-terminal helper domains, in addition to *NBS* domains, e.g., *Zf-BED, WRKY, BRX, LIM, DA1-like, Plant_tran, zf-RVT, Myb_DNA*-binding, AP2, DDE_Tnp_4, Thioredoxin, Jacalin, zf-BED, etc. These additional domains are known as immunological receptors with integrated domains (IDs) that resemble pathogen targets and are activated in response to effector change^74^. Previous literature data divided *NLRs* into two functional groups: (1) direct/indirect sensor *NLRs* that detect invasion and (2) helper *NLRs* that are genetically necessary for immunological activation by other *NLRs*^75,76^. Based on the presence and absence of the 2nd group, we have classified all 12,822 *NBS* genes into 168 classes. The number of different classes in different plant species also gives genetic features of land plants and their evolutions. Some of these identified classes are reported in the literature and well-characterized. As the *RNL* clade is usually characterized by a low copy number^77,78^, except Gymnosperms (there are 31 ADR homologs in spruce)^79^. RNLs exhibit extraordinary intron conservation in *Amborella* and dicots, which share four introns, but monocots have three introns (the second is missing)^80,81^. NRG and ADR were separated before angiosperms diverged and they are still retained in syntenic blocks across flowering plants and several lineages have lost NRG genes but not ADR genes^82^. The *TNLs* are divided into two subfamilies: (1) TIR1 and (2) *TIR2*^83^, while in some plants such as monocots, only TIR2 *NLRs* are maintained^83,84^. Many dicot species have *TIR1 NLRs,* however, other dicot lineages are devoid of them^85^. So, the presence and absence of these newly identified classes in our study further required functional characterization.

The evolutionary study of *NBS* genes in 33 land plants provided several common patterns of *NLR* evolution that were not visible in studies of a single species or plant family. A total of 664 orthogroups (OGs) were predicted among the land plants with only 8.2% of the total genes with unique sequences. Of these, we found many core and conserved OGs in plant species-specific lineage. The OG_00_ was shared with all plants except some lower plants, showing orthologs reduction due to their small and simple genome. The OG_2_ was another orthogroup that was found as the core orthogroup among land plants. The genome and tandem duplication events also cause the extension of OGs in the genome and cause genomic diversity^86,87^. The diversity analysis of *NBS* at the genic and genomics level also demonstrated duplication events at internal and terminal nodes of the phylogenetic tree during the evolutionary process..

The RNA-seq-based expression profiling of the *NBS* gene in *A. thaliana*, *Z. mays*, *G. hirsutum,* and *G. arboreum* demonstrates the significant role of the *NBS* gene in plant growth and development^88,89^. As in the control of *NLR* activity, gene transcription is an early regulatory step. To acquire resistance, proper *NLR* gene transcription is necessary as excessive transcription can cause programmed cell death, which is detrimental to plant growth and development. Overall expression profiling suggested that the OG_2_, OG_6_, and OG_15_ members showed the highest putative role in different tissues, under various biotic and abiotic stresses. The highest expression of these genes in the given plant species under biotic and abiotic stresses might be due to the presence of stress-responsive cis-regulatory elements in the promoter region, which was observed in the promoter sequences of *G. hirsutum* e.g. *Box, ARE, G-box, GT1-motif, ABRE, CGTCA-motif, TGACG-motif, TCT-motif, TCA- element, MBS, GATA-motif, CAT-box* and *O2*-site. In addition to the presence/absence of stress-responsive elements, epigenetic markers such as histone post-translational modifications and DNA methylation can also influence *NLR* gene expression by modulating their chromatin structure. *NLR* gene expression may be influenced by DNA methylation at the promoter, as the methylation in the promoter of the *Arabidopsis TNL* gene (MG1), upregulated *flg22* treatment, implying dynamic DNA methylation in *NLR* promoters during biotic stress. Additionally, the genetic variation in the UTRs, splicing sites, and exonic regions of *NBS* genes in the susceptible and tolerant *G. hirsutum* also depicted their role in the two lines under cotton leaf curl disease.

For long-term strategies to control disease in cotton and other important crops, the deployment of genomics tools including, NGS sequencing, exome sequencing, and genotype by sequencing, are helping to identify genetic variations (InDels, SNPs, SSR) associated with the functional diversity, which is further in marker-assisted selection (MAS) approach during breading of wild (resistant) and cultivars (susceptible)^46,69^. In the current study, we have made a comparison of genetic variation between a highly virus-resistant *G. hirsutum* accession and a highly susceptible cultivar and found several SNPs and InDels in tolerant accession. As breeding of virus-resistant cotton varieties with sufficient genetic diversity has been suggested as a durable strategy for controlling the disease effectively^90,91^ and the genetic basis of resistance and its inheritance are the key components for designing breeding strategies. In cotton plants, very little is known about the genetic variants associated with cotton leaf curl disease resistance. Therefore, our identified genetic variants might help develop CLCuD-resistant varieties, which is our next project.

Plant *NLR* proteins have been discovered to have a central *NBS* domain that resembles the AAA-ATPase family^92,93^. The ADP and ATP are the two molecules that have a putative role in the activation and deactivation of the *NBS* domain^94^. The binding of ADP with the *NBS* domain, causes deactivation, while its exchange with ATP opens the active site of the *NBS* domain and activates the molecular mechanism^95^. After activation, two intracellular proteins CC-*NLR* and *TIR-NLR* recognize viral effector molecules to activate the defense responses^96^. So, to undertrained the deep mechanism of *NBS* protein activation in cotton plants, we have presented a comparative molecular interaction of ADP and ATP with *NBS* proteins and found that the OG_2_ genes (*Gar01G01860*_OG2_*, Gar06G24920*_OG2_) showed more affinity to ATP molecule as compared to ADP molecule, suggesting their active role in defense response. Furthermore, the functional validation of OG_2_ genes (*Gar01G01860*_OG2_*, Gar06G24920*_OG2_), in *G. arboreum* (a naturally immune landrace) through virus-inducing gene silencing also increased the viral disease system in silenced plants as compared to control plants.

## Conclusion

The identification and analysis of resistance genes in plants have revealed interesting patterns of their evolution and diversification. The majority of these genes belong to the *NLR* receptor gene family, which can be traced back to the common ancestor of all green plants. The number and type of *NLR* genes vary greatly among different species, indicating their adaptation to cope with rapidly evolving pathogens. *NLRs* are frequently clustered in complex clusters, which may facilitate their diversification and development. Different classes of *NLRs* have been identified based on their N-terminal and C-terminal helper domains, some of which resemble pathogen targets and are activated in response to effector change. While the study of *NBS* genes in different plant species has revealed common patterns of evolution, the presence and absence of newly identified classes require further functional characterization. Furthermore, the *NBS* gene plays a significant role in plant growth and development, as demonstrated by RNA-seq expression profiling in various plant species. Proper transcription of the *NLR* gene is necessary for acquiring resistance, as excessive transcription can cause programmed cell death that is harmful to plant growth. The OG_2_, OG_6_, and OG_15_ members showed the highest putative role in different tissues, under various biotic and abiotic stresses, possibly due to stress-responsive cis-regulatory elements in the promoter region. Finally, the functional validation of OG_2_ (*Gar01G01860*_OG2_*, Gar06G24920*_OG2_) in resistant cotton plants demonstrated its putative role in plant resistance during cotton leaf curl disease infection. Overall, these findings provide insights into the genetic features and evolution of land plants.

### Supplementary Information

Supplementary Figures.

Supplementary Tables.

## Data Availability

Source data for all the graphs included in this paper are available as supplementary data.
