# Supplementary material for: Comparative analysis, diversification, and functional validation of plant nucleotide-binding site domain genes
Source: Sci Rep. 2024 May 24;14:11930. doi: 10.1038/s41598-024-62876-5 (PMC11126693; doi:10.1038/s41598-024-62876-5)
Supplement: Supplementary file 1 — Supplementary Figures. [file 41598_2024_62876_MOESM1_ESM.docx]

**Supplementary Materials**

Research Article

**Comparative Analysis, Diversification, and Functional Validation of Plant Nucleotide-Binding Site Domain Genes**

Athar Hussain^1, 2$*^, Aqsa Anwer Khan^3,^ Muhammad Qasim Aslam^1$^, Aquib Nazar^3^_,_ Nadir Zaman^3^, Ayesha Amin^4^, Muhammad Arslan Mahmood^5^, M. Shahid Mukhtar^6^, Hafiz Ubaid Ur Rahman^2^, Muhammed Farooq^1^, Muhammed Saeed^7^, Imran Amin^1^*, Shahid Mansoor^1,8^*

^1^National Institute for Biotechnology and Genetic Engineering, College of Pakistan Institute of Engineering and Applied Sciences (PIEAS), Faisalabad, 38000, Pakistan

^2^Genomics Lab, School of Food and Agricultural Sciences (SFAS), University of Management and Technology (UMT), Lahore, 54000, Pakistan

^3^Department of Life Science, University of Management and Technology (UMT), Lahore, 54000, Pakistan

^4^Department of Biological Sciences, Superior University, Lahore, 54000, Pakistan

^5^Plant Science Division, Research School of Biology, The Australian National University, Canberra, ACT 2601, Australia

^6^Department of Biology, University of Alabama at Birmingham, 1300 University Blvd. Birmingham, AL 35294, USA.

^7^Rheinland-Pfälzische Technische Universität Kaiserslautern-Landau Abteilung Phytopathologie, Paul-Ehrlich-Straße 22, 67653 Kaiserslautern, Germany.

^8^Jamil ur Rehman Center for Genome Research, International Center for Chemical and Biological Sciences, University of Karachi, Karachi, 74000, Pakistan

^$^ Equal contribution

^*^Corresponding authors:imranamin1@yahoo.com, shahidmansoor7@gmail.com atharmutahari@gmail.com

**Keywords:** Genome-wide, NLR, Diversity, Land Plants, evolution, classification, VIGS, Expression


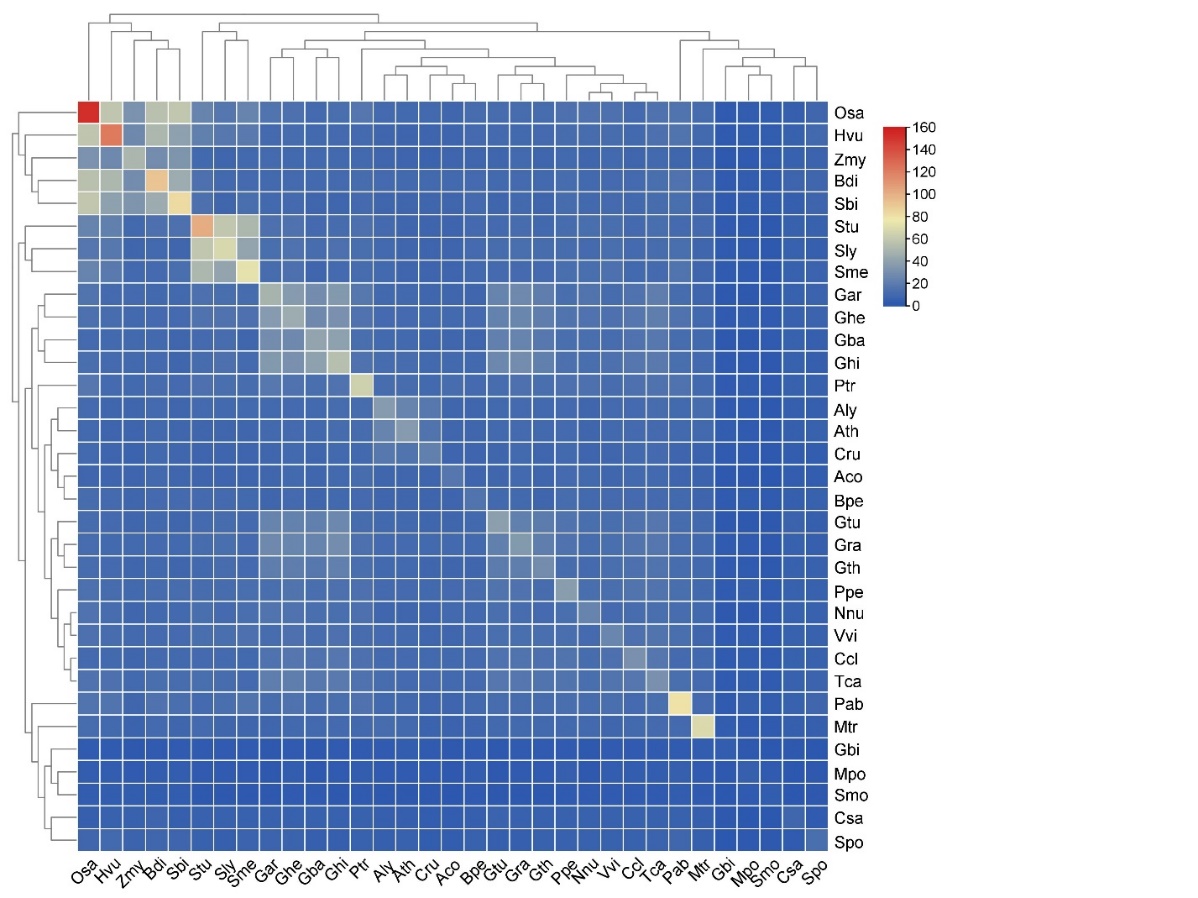


**Figure S1** Orthogroups overlapping and sharing among plant species. The heat map was generated using TBTools [1].


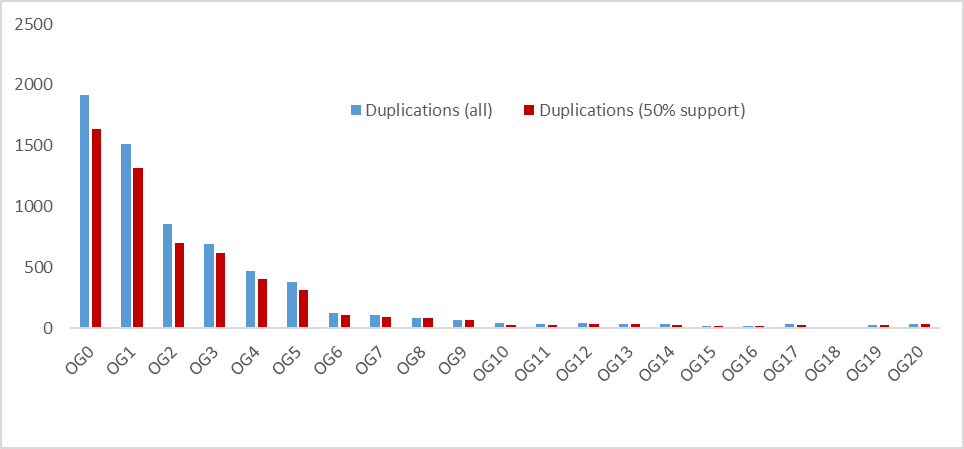


**Figure S2** List of orthogroups and their duplication events at 100% and at 50% probability value.


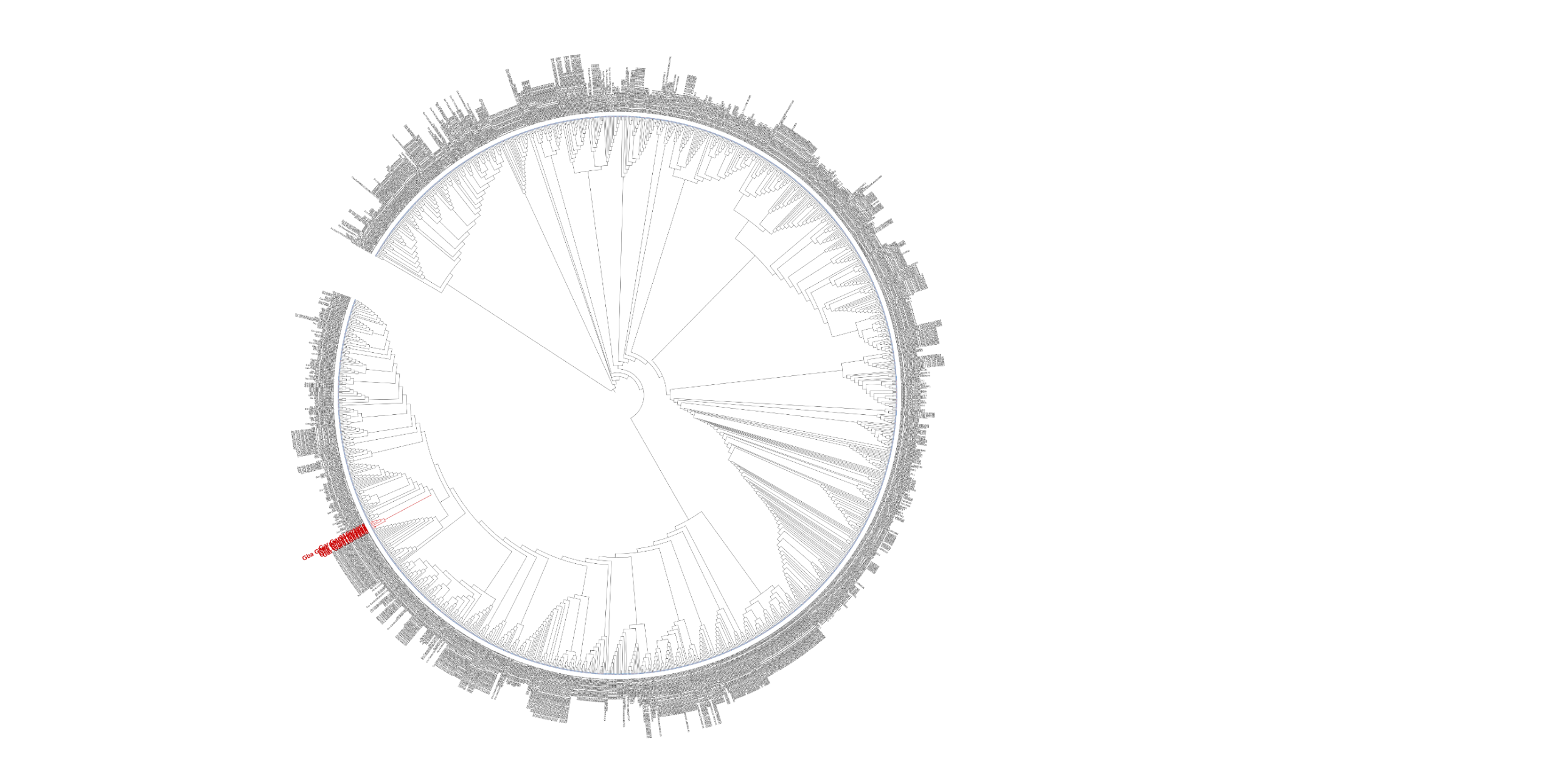


**Figure S3 .** Phylogenetic tree of orthogroup 2 (OG_2_). The red-colored genes are the functionally validated genes in cotton. Tree was generated using FastME 2.0 version[2].


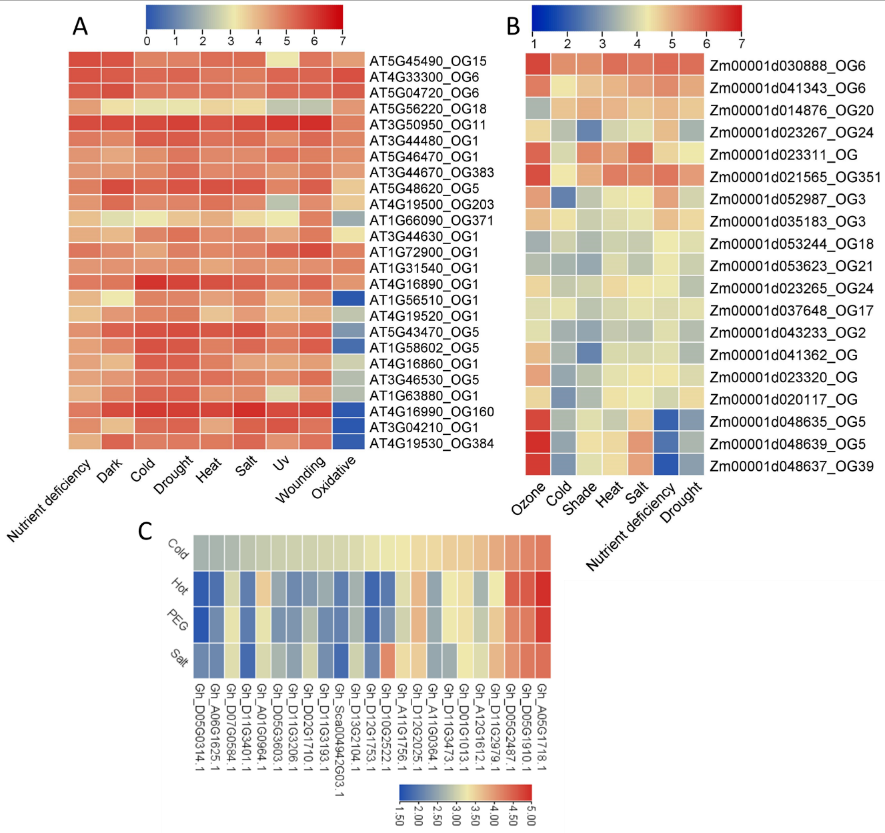


**Figure S4** RNA-seq-based expression profiling of NBS gene under various abiotic stresses. The OGs associated with gene IDs represent the Orthogroups. A) A. thaliana, B) Z. mays C) G. hirsutum. Heat maps were generated using TBTools[1].


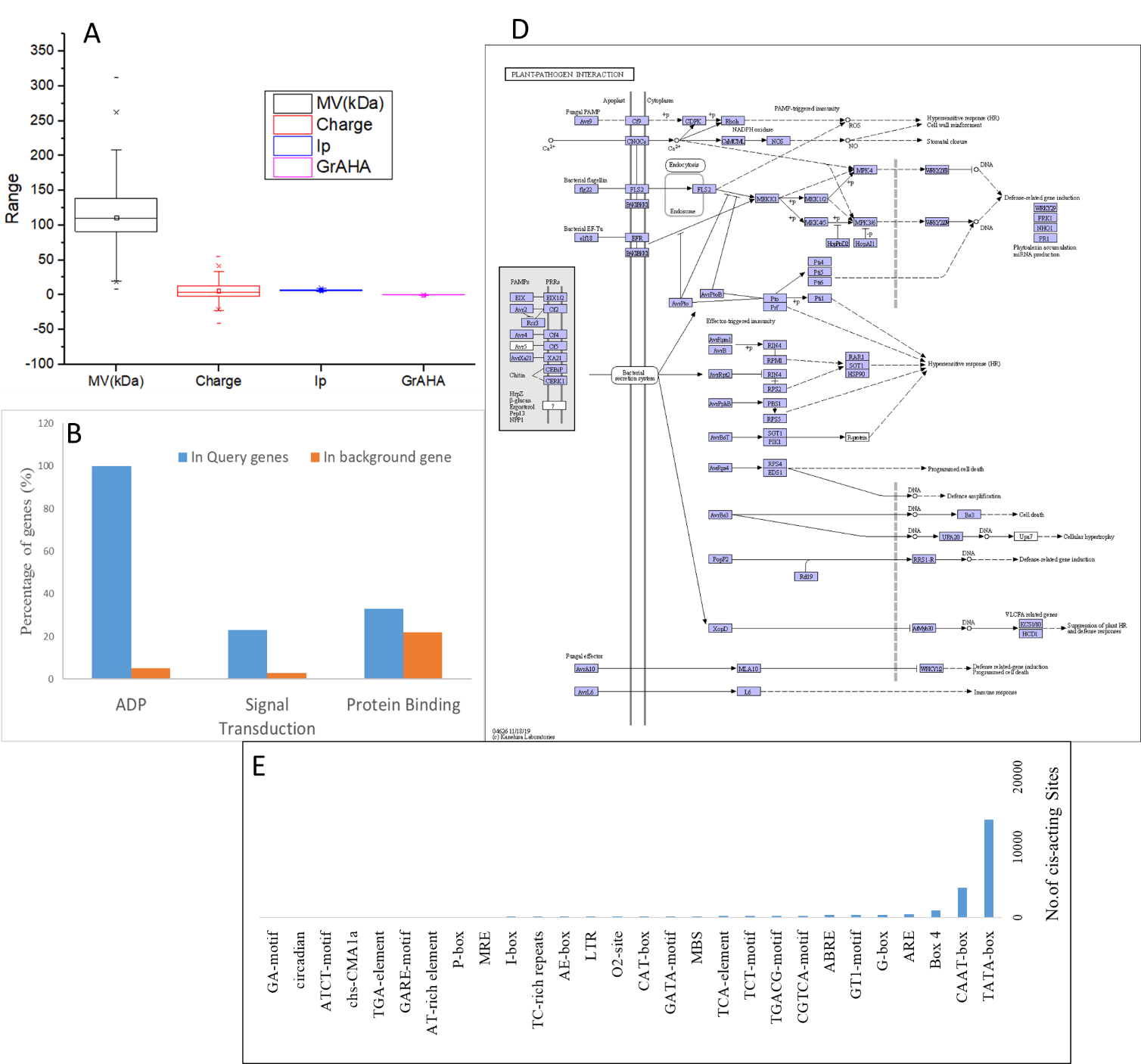


**Figure S5** Genetic annotation of NBS genes. A) physio chemistry of *GhNBS* proteins, B) molecular function enrichment of *GhNBS*, C) Predicted metabolic pathway using KEGG-based pathway analysis ([www.kegg.jp/kegg/kegg1.html](http://www.kegg.jp/kegg/kegg1.html) ), and D) *Cis*-regulatory elements found in the promoter region of *GhNBS* genes. The graphs were generated in MS Office Excel, and the pathway was extracted from KEGG. The license of the KEGG pathway is also attached with the additional file.


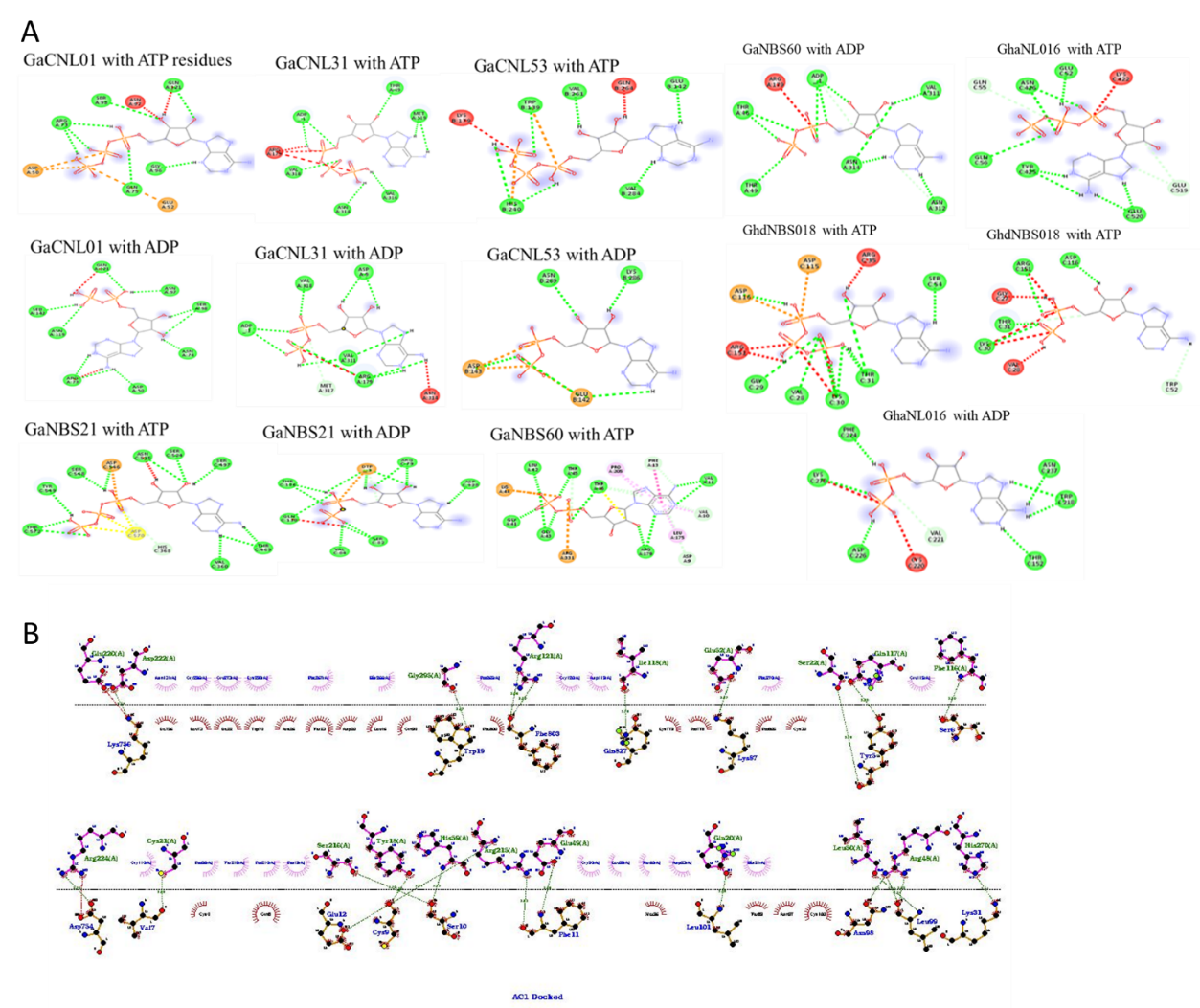


**Figure S6** Molecular docking of Nucleotide-binding site proteins. A) 2D graph of docking of NBS proteins with ATP and ADP, B) 2D interacting residues of NBS proteins with AC1 viral proteins. The 2D interacting figures are generated using BIOVIA Discovery Studio Visualizer 4.5[3].


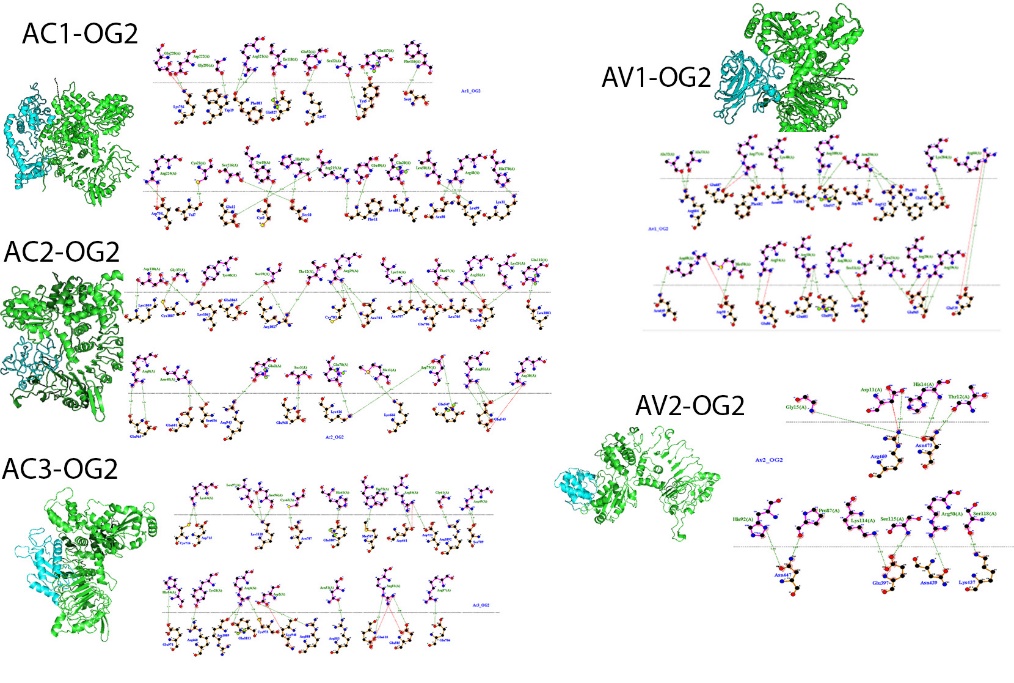


**Figure S7** Host-pathogen protein-protein interaction. The Begomoviral proteins and their interaction with OG_2_ (Gar06G24920_OG2) proteins in cotton. The 3D structure presented; Cyan=viral, green=host (OG2). The 2D graph represented the interacting residues of host and pathogen proteins. The 2D interacting figures generated using LigPlot+ Version 2.2[4].


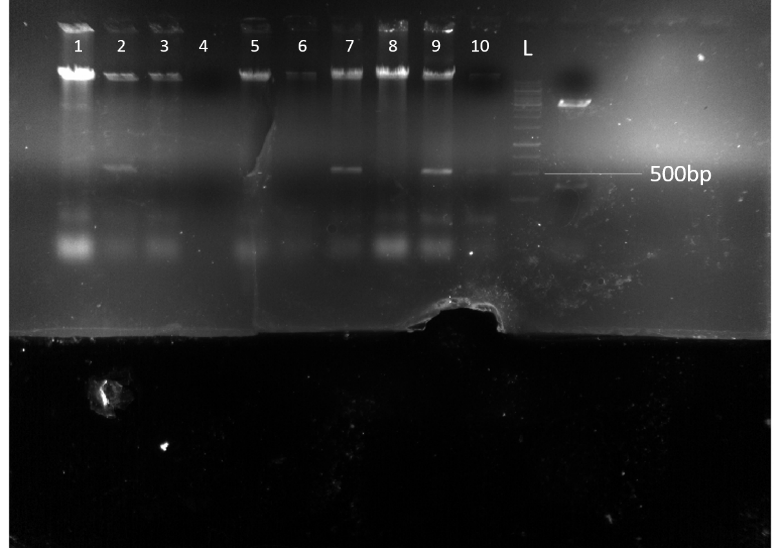


**Figure S8** Cloning of G2 gene in VIGS vector. Lanes 1-10 represent restriction digestion TRV: G2 clone. pTRV2 vector representing the size of ～10 kb while the fragment of size ～500bp are representing G2 gene. L is a ladder of size 1KB.


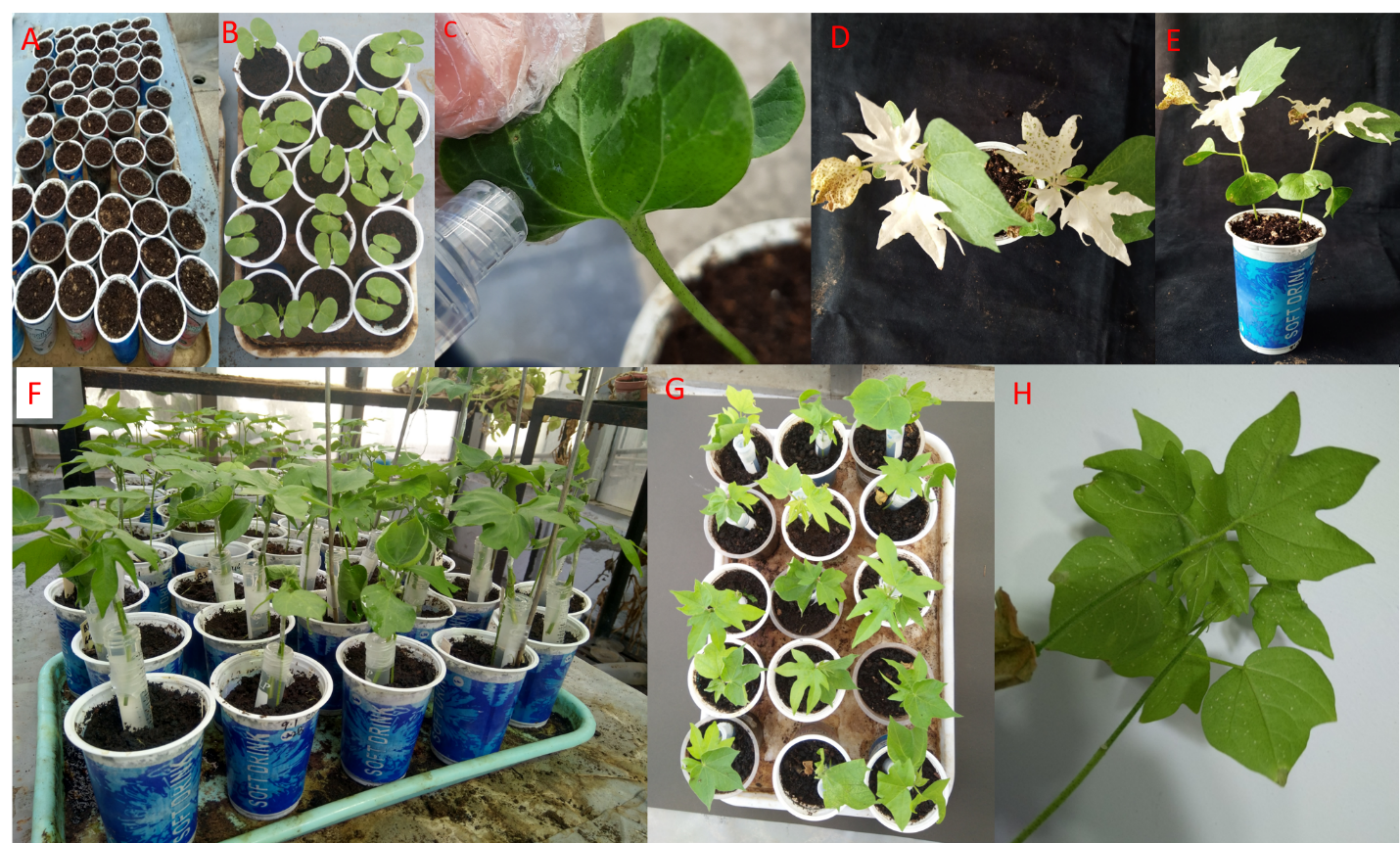


**Figure S9** CLCuD inoculation and estimation of virus titer. Panels A, B and C show the sowing, germination and inoculation of cotton plants at the cotyledonary leaf stage. Panel D depicts a completely beached phenotype of TRV: GrCLA1 inoculated G. arboreum plants. F and G are showing graft-mediated inoculation of CLCuD in VIGS plants of G. arboreum while H is showing whitefly-mediated inoculation.

**References of software/tools:**

[1] C. Chen, H. Chen, Y. Zhang, H. R. Thomas, M. H. Frank, Y. He*, et al.*, "TBtools: an integrative toolkit developed for interactive analyses of big biological data," *Molecular Plant,* vol. 13, pp. 1194-1202, 2020.

[2] V. Lefort, R. Desper, and O. Gascuel, "FastME 2.0: a comprehensive, accurate, and fast distance-based phylogeny inference program," *Molecular biology and evolution,* vol. 32, pp. 2798-2800, 2015.

[3] D. Studio, "Discovery studio," *Accelrys [2.1],* 2008.

[4] R. A. Laskowski and M. B. Swindells, "LigPlot+: multiple ligand–protein interaction diagrams for drug discovery," ed: ACS Publications, 2011.
